# Supplementary figures and images for: SNARE protein USE1 is involved in the glycosylation and the expression of mumps virus fusion protein and important for viral propagation
Source: PLoS Pathog. 2022 Dec 8;18(12):e1010949. doi: 10.1371/journal.ppat.1010949 (PMC9731409; doi:10.1371/journal.ppat.1010949)

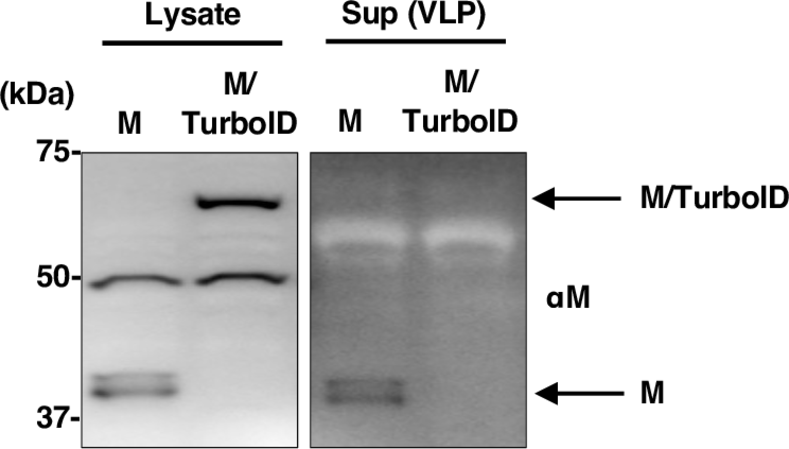

Supplement: S1 Fig — VLP assay showing the amounts of the wildtype M or the M/TurboID proteins expressed in the cells (Lysate) or released into the supernatants (Sup). (TIF) [file ppat.1010949.s001.tif]

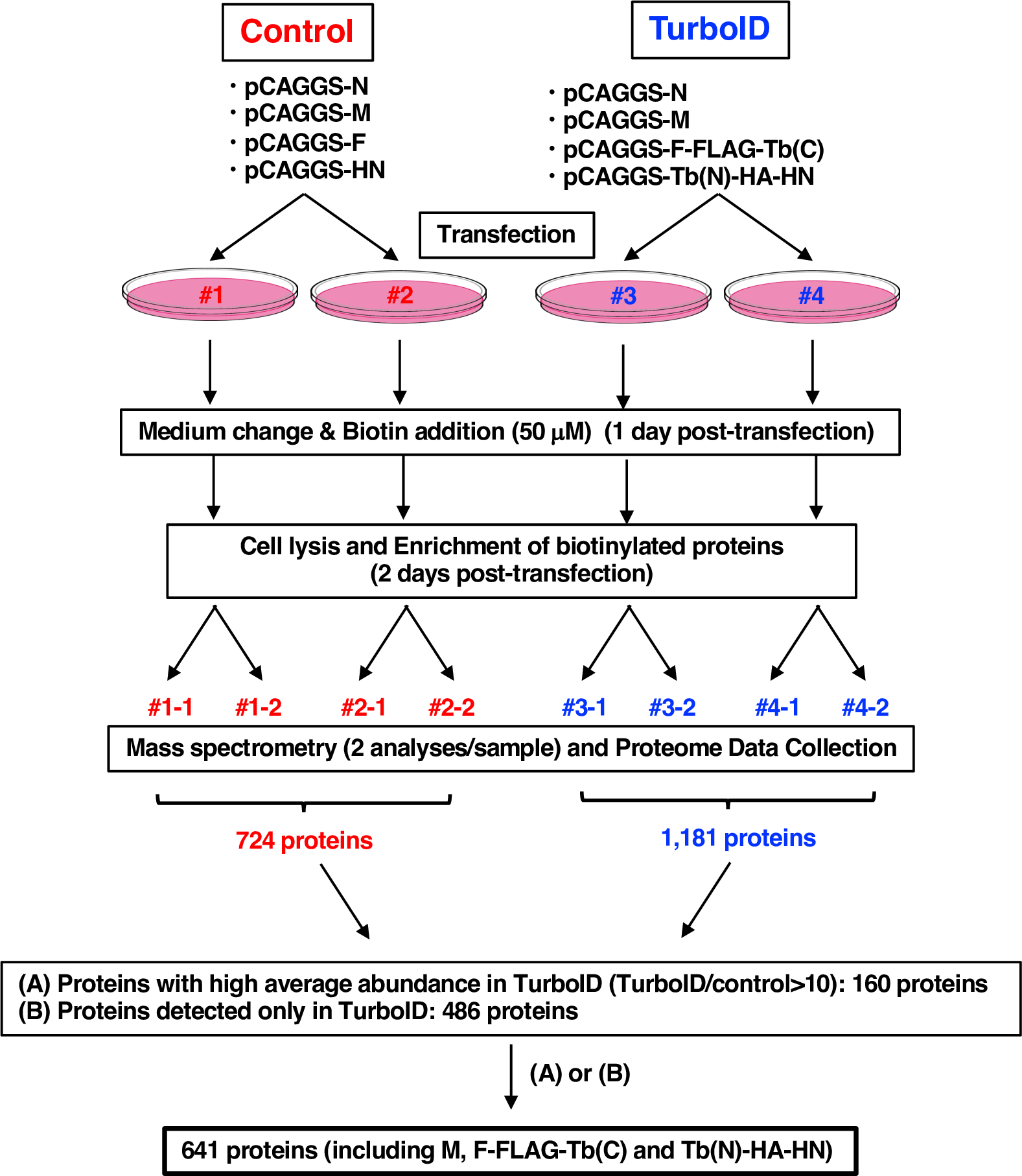

Supplement: S2 Fig — 293T cells transfected with the indicated plasmids were labeled with 50 μM biotin. The cells were lysed, and biotinylated proteins were enriched using streptavidin beads, digested into peptides, and analyzed by LS-MS/MS. MS analysis was performed twice per sample. The averages of the detection intensities (protein abundances) obtained from each sample (control and TurboID) were calculated. Factors that were detected only in the split-TurboID sample or factors with an intensity ratio (TurboID/control) of ≥10-fold were then extracted, and a total of 641 proteins were identified as being significantly enriched in the split-TurboID-expressing cells. (TIF) [file ppat.1010949.s002.tif]

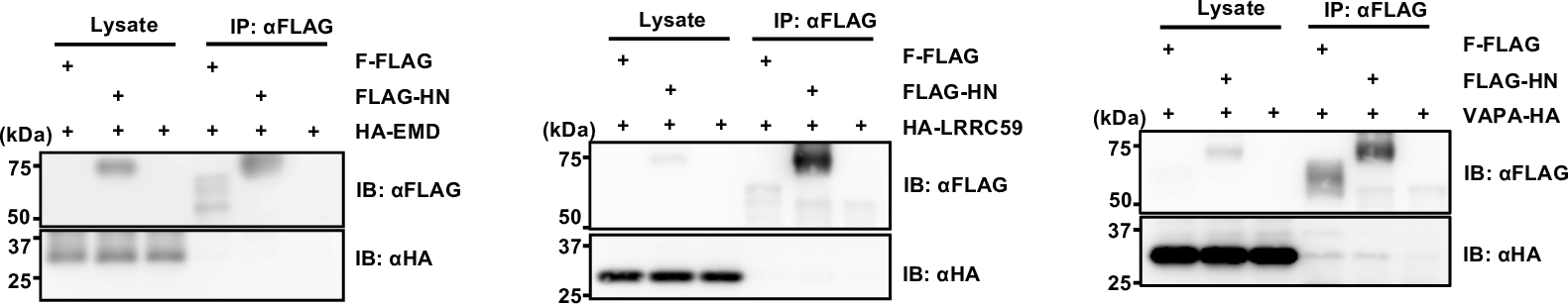

Supplement: S3 Fig — Immunoprecipitation assay showing the interaction of F-FLAG or FLAG-HN proteins with HA-EMD (left), HA-LRRC59 (center), or VAPA-HA (right) in 293T cells. (TIF) [file ppat.1010949.s003.tif]

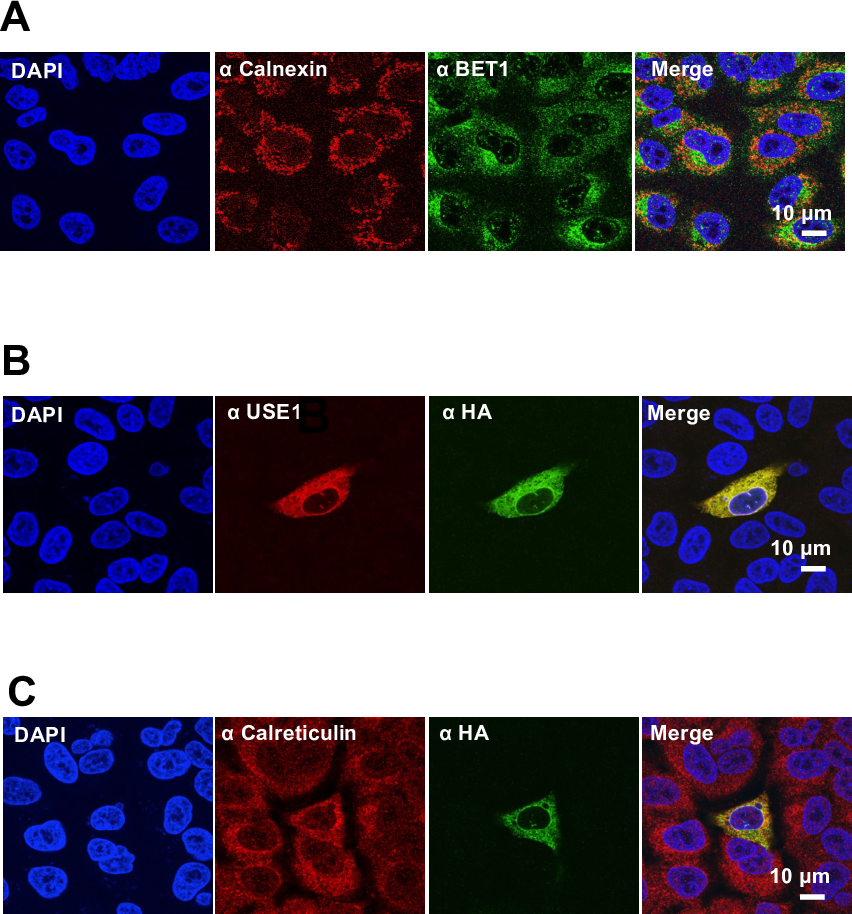

Supplement: S4 Fig — (A) Immunofluorescence of A549 cells treated with anti-Calnexin (red) and anti-BET (green) antibodies. (B) Immunofluorescence of A549 cells expressing HA-USE1 treated with anti-USE1 (red) and anti-HA (green) antibodies. (C) Immunofluorescence of A549 cells expressing HA-USE1 treated with anti-Calreticulin (red) and anti-HA (green) antibodies. (TIF) [file ppat.1010949.s004.tif]

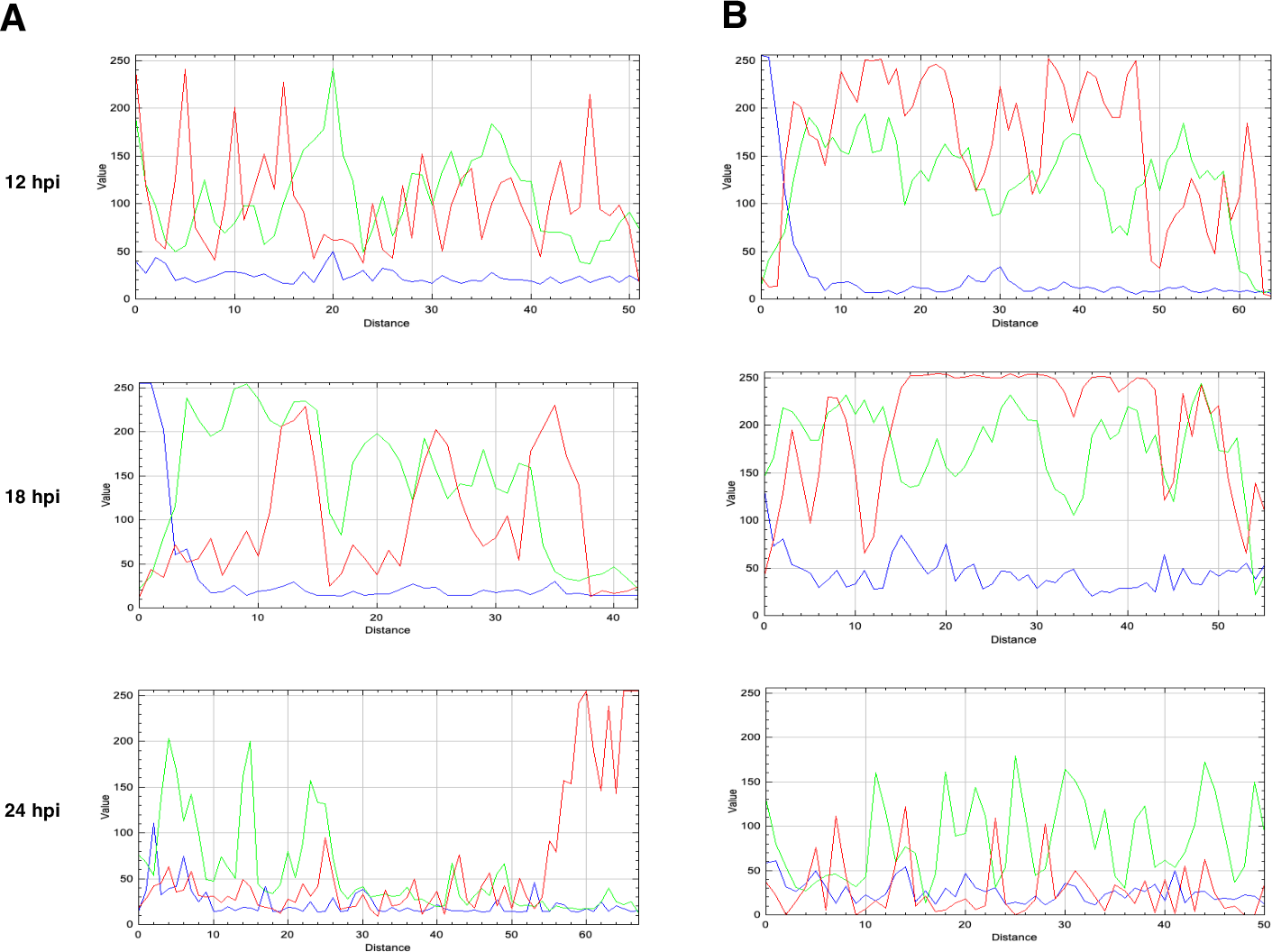

Supplement: S5 Fig — (A–B) RGB line profiles along the lines shown in Fig 4B (A) and 4C (B) were analyzed by using ImageJ software (Version: 2.3.0). (TIF) [file ppat.1010949.s005.tif]
