# Supplementary material for: SNARE protein USE1 is involved in the glycosylation and the expression of mumps virus fusion protein and important for viral propagation
Source: PLoS Pathog. 2022 Dec 8;18(12):e1010949. doi: 10.1371/journal.ppat.1010949 (PMC9731409; doi:10.1371/journal.ppat.1010949)
Supplement: S1 Table — (PDF) [file ppat.1010949.s006.pdf]

S1 Table. List of proteins identified to be enriched in the split-TurboID-expressed cells.

Accession	Description	MW [kDa]	Entrez Gene ID	Gene Symbol	Number of peptides	Aboundance ratio (TurboID/Control)	Aboundance (Control)	Abundance (TurboID)	Number of peptides found (Control)	Number of peptides found (TurboID)
P37802-2	Isoform 2 of Transgelin-2 [OS=Homo sapiens]	24.4	8407	TAGLN2	16	9137.063476	1855.4545	16953405.54	0	4
Q7Z2W4	zinc finger CCCH-type antiviral protein 1 [OS=Homo sapiens]	101.4	56829	ZC3HAV1	37	8993.184623	3610.325745	32468325.97	0	4
P30519	Heme oxygenase 2 [OS=Homo sapiens]	36	3163	HMOX2	18	7964.707187	3703.328027	29495923.36	0	4
P50402	Emerin [OS=Homo sapiens]	29	2010	EMD	24	7813.048964	3402.903206	26587049.37	0	4
O14967	calmegin [OS=Homo sapiens]	70	1047	CLGN	21	6239.554415	1624.528163	10136331.87	0	4
P29966	Myristoylated alanine-rich C-kinase substrate [OS=Homo sapiens]	31.5	4082	MARCKS	8	5780.56108	1053.88477	6092045.282	0	4
P51572-2	Isoform 2 of B-cell receptor-associated protein 31 [OS=Homo sapiens]	34.7	10134	BCAP31	21	4699.778115	24291.69582	114165580.4	0	4
MuV_Odate_TbN-HA-HN	MuV_Odate_TbN-HA-HN protein	73.5			26	3770.819802	59599.81019	224740144.5	0	4
Q9H3N1	Thioredoxin-related transmembrane protein 1 [OS=Homo sapiens]	31.8	81542	TMX1	18	3377.052586	7797.401034	26332233.32	0	4
P08240-1	signal recognition particle receptor subunit alpha [OS=Homo sapiens]	69.8	6734	SRPR; SRPRA	36	3054.303697	16760.31987	51191106.95	0	4
O15498	Synaptobrevin homolog YKT6 [OS=Homo sapiens]	22.4	10652	YKT6	9	2894.620967	1620.340642	4690271.997	0	4
P49069	calcium signal-modulating cyclophilin ligand [OS=Homo sapiens]	32.9	819	CAMLG	12	2418.481652	1311.339238	3171449.886	0	3
P49959-3	Isoform 3 of Double-strand break repair protein MRE11 [OS=Homo sapiens]	81	4361	MRE11A; MRE11	31	1726.677278	5147.367967	8887843.309	0	4
P15170-3	Isoform 3 of Eukaryotic peptide chain release factor GTP-binding subunit ERF3A [OS=Homo sapiens]	68.7	2935	GSPT1	25	1560.220931	14578.13913	22745117.8	0	4
Q6PL24	Protein TMED8 [OS=Homo sapiens]	35.7	283578	TMED8	8	1256.006309	1786.747518	2244166.155	0	4
Q9Y2H6	Fibronectin type-III domain-containing protein 3a [OS=Homo sapiens]	131.8	22862	FNDC3A	23	1161.230986	17130.96289	19893004.93	0	1
P18031	Tyrosine-protein phosphatase non-receptor type 1 [OS=Homo sapiens]	49.9	5770	PTPN1	25	1092.199908	22099.58886	24137168.92	0	4
O15155	BET1 homolog [OS=Homo sapiens]	13.3	10282	BET1	7	1011.773001	3606.445397	3648904.081	0	4
P78344-1	Eukaryotic translation initiation factor 4 gamma 2 [OS=Homo sapiens]	102.3	1982	EIF4G2	21	1002.784507	4354.871148	4366997.318	0	4
P27824-2	Isoform 2 of Calnexin [OS=Homo sapiens]	71.5	821	CANX	17	959.9030097	13343.47925	12808445.89	0	4
Q86UE4	protein LYRIC [OS=Homo sapiens]	63.8	92140	MTDH	43	838.0296005	150001.6211	125705798.6	0	4
Q92575	UBX domain-containing protein 4 [OS=Homo sapiens]	56.7	23190	UBXN4	17	811.3812808	25211.98681	20456534.15	0	4
P10599-1	thioredoxin [OS=Homo sapiens]	11.7	7295	TXN	7	800.417828	61060.8234	48874171.65	0	4
P46821	microtubule-associated protein 1B [OS=Homo sapiens]	270.5	4131	MAP1B	14	788.0062637	1660.216527	1308261.022	0	4
O15027-5	Isoform 5 of Protein transport protein Sec16A [OS=Homo sapiens]	235.6	9919	SEC16A	20	774.6467468	5621.051584	4354329.323	1	4
Q9P246	Stromal interaction molecule 2 [OS=Homo sapiens]	83.9	57620	STIM2	14	770.3256592	5857.009271	4511804.528	0	4
Q8N0X7	Spartin [OS=Homo sapiens]	72.8	23111	SPG20; SPART	10	706.8464005	3263.060693	2306482.706	0	4
Q6MZT1	Regulator of G-protein signaling 7-binding protein [OS=Homo sapiens]	28.9	401190	RGS7BP	1	674.5323869	1006.842888	679148.1363	0	4
Q96KC8	DnaJ homolog subfamily C member 1 [OS=Homo sapiens]	63.8	64215	DNAJC1	15	663.1725668	13953.7395	9253737.244	0	4
Q15907	Ras-related protein Rab-11B [OS=Homo sapiens]	24.5	9230	RAB11B	6	642.3473001	2078.344059	1335018.695	0	4
Q92900	Regulator of nonsense transcripts 1 [OS=Homo sapiens]	124.3	5976	UPF1	14	640.5483965	3287.153555	2105580.939	0	3
Q9NRY5	Protein FAM114A2 [OS=Homo sapiens]	55.4	10827	FAM114A2	21	551.2857426	8541.391353	4708747.275	0	2
P42167	Lamina-associated polypeptide 2, isoforms beta/gamma [OS=Homo sapiens]	50.6	7112	TMPO	49	549.0952864	710166.8644	389949277.8	0	4
Q86XL3-1	ankyrin repeat and LEM domain-containing protein 2 [OS=Homo sapiens]	104.1	23141	ANKLE2	24	547.6776248	10571.7877	5789931.578	0	4
Q86UP2-1	Kinectin [OS=Homo sapiens]	156.2	3895	KTN1	99	532.035347	220220.1341	117164895.4	0	4
Q13586	stromal interaction molecule 1 [OS=Homo sapiens]	77.4	6786	STIM1	35	520.0825237	79864.9876	41536384.31	0	4
Q15417	Calponin-3 [OS=Homo sapiens]	36.4	1266	CNN3	7	510.427627	1891.939619	965698.2501	0	4
P43487	Ran-specific GTPase-activating protein [OS=Homo sapiens]	23.3	5902	RANBP1	6	504.159517	3266.49242	1646833.241	0	2
Q9BSJ8-2	Isoform 2 of Extended synaptotagmin-1 [OS=Homo sapiens]	123.9	23344	ESYT1	16	503.3151558	9242.264062	4651771.576	0	4
Q9Y266	nuclear migration protein nudC [OS=Homo sapiens]	38.2	10726	NUDC	18	481.7403332	24592.20953	11847059.21	0	4
Q9HBM0-1	vezatin [OS=Homo sapiens]	88.6	55591	VEZT	11	466.699443	5305.640626	2476139.525	0	4
Q5T8D3-1	Acyl-CoA-binding domain-containing protein 5 [OS=Homo sapiens]	60.1	91452	ACBD5	15	460.568295	6801.152139	3132395.044	0	4
Q9NZ43-1	Vesicle transport protein USE1 [OS=Homo sapiens]	29.4	55850	USE1	13	454.725617	13132.31338	5971599.302	0	4
Q9H089	Large subunit GTPase 1 homolog [OS=Homo sapiens]	75.2	55341	LSG1	23	433.0331021	17440.7678	7552429.784	0	4
Q96HC4	PDZ and LIM domain protein 5 [OS=Homo sapiens]	63.9	10611	PDLIM5	4	426.6608335	1436.602509	612942.024	0	4
Q9P2W9	Syntaxin-18 [OS=Homo sapiens]	38.7	53407	STX18	10	383.2299954	5648.900262	2164828.022	0	4
Q15006	ER membrane protein complex subunit 2 [OS=Homo sapiens]	34.8	9694	EMC2	9	347.7078378	2971.612971	1033253.121	0	4
Q9UQN3-1	Charged multivesicular body protein 2b [OS=Homo sapiens]	23.9	25978	CHMP2B	5	345.9282135	4760.848495	1646911.814	0	4
Q9P2E9-1	Ribosome-binding protein 1 [OS=Homo sapiens]	152.4	6238	RRBP1	38	334.069353	75583.87157	25250255.07	0	4
P16949-1	Stathmin [OS=Homo sapiens]	17.3	3925	STMN1	8	333.3561279	18698.61543	6233298.038	0	4
Q96C24	Synaptotagmin-like protein 4 [OS=Homo sapiens]	76	94121	SYTL4	15	323.1838586	9287.136113	3001452.484	0	1
Q05682-4	Isoform 4 of Caldesmon [OS=Homo sapiens]	62.6	800	CALD1	8	314.7157874	4326.068795	1361482.147	0	4
P31948	stress-induced-phosphoprotein 1 [OS=Homo sapiens]	62.6	10963	STIP1	13	308.4744719	7452.161497	2298801.582	0	4
P16435	NADPH--cytochrome P450 reductase [OS=Homo sapiens]	76.6	5447	POR	15	296.7366518	10353.27998	3072197.635	0	4
O00151	PDZ and LIM domain protein 1 [OS=Homo sapiens]	36	9124	PDLIM1	8	286.0630225	5364.771752	1534662.822	0	4
P07355-2	Isoform 2 of Annexin A2 [OS=Homo sapiens]	40.4	302	ANXA2	11	263.8529862	9106.813956	2402860.057	0	4
P15311	Ezrin [OS=Homo sapiens]	69.4	7430	EZR	20	262.1160109	16933.25499	4438477.249	0	4
MuV_Odate_F-FLAG-TbC	MuV_Odate_F-FLAG-TbC protein	87.3			34	255.0570759	51255.68058	13073124.01	0	4
Q9NRZ9	Lymphoid-specific helicase [OS=Homo sapiens]	97	3070	HELLS	21	254.9604195	30337.52765	7734868.776	0	4
Q13442	28 kDa heat- and acid-stable phosphoprotein [OS=Homo sapiens]	20.6	11333	PDAP1	11	240.1606846	26987.88796	6481429.648	0	4
Q7KZF4	staphylococcal nuclease domain-containing protein 1 [OS=Homo sapiens]	101.9	27044	SND1	7	233.4742173	2635.884131	615410.9845	0	4
Q9Y2U8	inner nuclear membrane protein Man1 [OS=Homo sapiens]	99.9	23592	LEMD3	12	215.1162411	7630.426901	1641428.753	0	2
P50990	T-complex protein 1 subunit theta [OS=Homo sapiens]	59.6	10694	CCT8	34	207.2115244	116470.7521	24134082.09	0	3
Q9Y394	Dehydrogenase/reductase SDR family member 7 [OS=Homo sapiens]	38.3	51635	DHRS7	7	201.1062057	13561.74902	2727351.888	0	4
Q13190	Syntaxin-5 [OS=Homo sapiens]	39.6	6811	STX5	14	195.6095943	55297.75014	10816770.47	0	4
P11142-1	Heat shock cognate 71 kDa protein [OS=Homo sapiens]	70.9	3312	HSPA8	39	190.9941958	290937.4091	55567356.47	0	4
P62316	Small nuclear ribonucleoprotein Sm D2 [OS=Homo sapiens]	13.5	6633	SNRPD2	5	189.498474	15794.24114	2992984.594	0	4
Q9Y490	Talin-1 [OS=Homo sapiens]	269.6	7094	TLN1	33	176.014405	20675.86392	3639249.887	0	4
Q96TC7	Regulator of microtubule dynamics protein 3 [OS=Homo sapiens]	52.1	55177	FAM82A2; RMDN3	3	174.7536844	1432.207555	250283.5469	0	4
Q9HDC5	junctophilin-1 [OS=Homo sapiens]	71.6	56704	JPH1	13	144.5608175	38607.52766	5581135.762	0	4
P40855-1	Peroxisomal biogenesis factor 19 [OS=Homo sapiens]	32.8	5824	PEX19	4	141.6631343	1770.918608	250873.8806	0	4
Q5VV42	threonylcarbamoyladenosine tRNA methylthiotransferase [OS=Homo sapiens]	65.1	54901	CDKAL1	20	138.1816295	49412.97785	6827965.795	0	4
Q01968-1	Inositol polyphosphate 5-phosphatase OCRL-1 [OS=Homo sapiens]	104.1	4952	OCRL	9	134.8899771	10093.03214	1361448.874	0	4
P61011-1	signal recognition particle 54 kDa protein [OS=Homo sapiens]	55.7	6729	SRP54	34	133.2925396	249963.0585	33318210.86	0	4
Q96A33-1	Coiled-coil domain-containing protein 47 [OS=Homo sapiens]	55.8	57003	CCDC47	21	124.6192138	321273.1139	40036802.86	0	4
P17980	26S proteasome regulatory subunit 6A [OS=Homo sapiens]	49.2	5702	PSMC3	11	124.1930593	10623.44504	1319358.14	0	4
Q9NR31	GTP-binding protein SAR1a [OS=Homo sapiens]	22.4	56681	SAR1A	5	123.9973076	12452.74859	1544107.298	0	4
A8CG34-1	Nuclear envelope pore membrane protein POM 121C [OS=Homo sapiens]	125	100101267	POM121C	13	117.9558627	47467.30169	5599046.521	0	4
Q8NFH5	Nucleoporin NUP53 [OS=Homo sapiens]	34.8	129401	NUP35	7	117.1272327	8010.194547	938211.9208	0	4
O15173-2	Isoform 2 of Membrane-associated progesterone receptor component 2 [OS=Homo sapiens]	26.2	10424	PGRMC2	7	113.981112	6415.422106	731236.9457	2	4
P23588	eukaryotic translation initiation factor 4B [OS=Homo sapiens]	69.1	1975	EIF4B	23	113.0388161	165655.8415	18725540.2	0	4
P0DMV8	heat shock 70 kDa protein 1A [OS=Homo sapiens]	70	3304; 3303	HSPA1B; HSPA1A	40	111.0659592	531787.6265	59063502.83	1	4
P10636-2	Isoform Fetal-tau of Microtubule-associated protein tau [OS=Homo sapiens]	36.7	4137	MAPT	4	108.6578296	3203.04714	348036.1503	0	3
O00264	Membrane-associated progesterone receptor component 1 [OS=Homo sapiens]	21.7	10857	PGRMC1	6	105.2139216	17396.62308	1830366.937	0	4
P49327	Fatty acid synthase [OS=Homo sapiens]	273.3	2194	FASN	11	103.8806017	6556.533529	681096.6483	0	4
Q9H4M9	EH domain-containing protein 1 [OS=Homo sapiens]	60.6	10938	EHD1	6	103.5690008	3317.122909	343551.1051	0	4
Q16850-1	lanosterol 14-alpha demethylase [OS=Homo sapiens]	56.8	1595	CYP51A1	9	103.1364309	5015.422475	517272.7735	0	4
P30419	glycylpeptide N-tetradecanoyltransferase 1 [OS=Homo sapiens]	56.8	4836	NMT1	2	101.4536345	3481.116877	353171.9591	0	4
Q9NPA0	ER membrane protein complex subunit 7 [OS=Homo sapiens]	26.5	56851	EMC7	6	97.28201506	22292.81249	2168689.72	0	4
Q8NDI1-2	Isoform 2 of EH domain-binding protein 1 [OS=Homo sapiens]	136.2	23301	EHBP1	21	96.50804207	55314.5135	5338295.396	0	4
Q14247-1	Src substrate cortactin [OS=Homo sapiens]	61.5	2017	CTTN	16	95.53849596	191782.5043	18322612.01	0	3
Q92598-4	Isoform 4 of Heat shock protein 105 kDa [OS=Homo sapiens]	97.4	10808	HSPH1	13	95.08928498	19348.66004	1839850.249	0	4
O95359	Transforming acidic coiled-coil-containing protein 2 [OS=Homo sapiens]	309.2	10579	TACC2	7	94.88708046	17915.01751	1699903.708	0	4
P52429	Diacylglycerol kinase epsilon [OS=Homo sapiens]	63.9	8526	DGKE	8	94.8660165	4500.06957	426903.674	0	4
O95721	Synaptosomal-associated protein 29 [OS=Homo sapiens]	29	9342	SNAP29	15	92.24882234	97692.16196	9011986.893	0	4
Q6NUQ4	Transmembrane protein 214 [OS=Homo sapiens]	77.1	54867	TMEM214	17	91.04937709	47347.71195	4310979.68	0	4
Q96KG9	N-terminal kinase-like protein [OS=Homo sapiens]	89.6	57410	SCYL1	13	88.99139461	13786.7131	1226898.826	0	4
P49006	MARCKS-related protein [OS=Homo sapiens]	19.5	65108	MARCKSL1	3	87.85915374	90963.13915	7991944.427	0	4
P49790	Nuclear pore complex protein Nup153 [OS=Homo sapiens]	153.8	9972	NUP153	34	86.59134357	135520.9751	11734943.31	0	4
Q9UJY5-6	Isoform 6 of ADP-ribosylation factor-binding protein GGA1 [OS=Homo sapiens]	72.2	26088	GGA1	6	83.22867134	5557.010924	462502.6358	0	4
P16615	Sarcoplasmic/endoplasmic reticulum calcium ATPase 2 [OS=Homo sapiens]	114.7	488	ATP2A2	5	79.65180555	5018.926483	399766.5564	0	2
P35606	Coatomer subunit beta' [OS=Homo sapiens]	102.4	9276	COPB2	10	77.22615922	39954.02485	3085495.885	0	4
Q96HA1-1	Nuclear envelope pore membrane protein POM 121 [OS=Homo sapiens]	127.6	9883	POM121	12	76.50790034	1411.39674	107983.0011	0	4
P48668	Keratin, type II cytoskeletal 6C [OS=Homo sapiens]	60	286887; 3853	KRT6C; KRT6A	13	72.44836769	29436.29384	2132611.44	0	4
Q96AG4	Leucine-rich repeat-containing protein 59 [OS=Homo sapiens]	34.9	55379	LRRC59	19	70.35535205	413201.8436	29070961.17	0	4
P51149	ras-related protein Rab-7a [OS=Homo sapiens]	23.5	7879	RAB7A	5	67.00511809	24524.50332	1643267.241	0	4
O95373	Importin-7 [OS=Homo sapiens]	119.4	10527	IPO7	3	66.55462177	4295.371628	285876.834	0	4
Q9Y6Y8	SEC23-interacting protein [OS=Homo sapiens]	111	11196	SEC23IP	15	65.95341018	55669.8465	3671616.221	0	3
Q14318-2	Isoform 2 of Peptidyl-prolyl cis-trans isomerase FKBP8 [OS=Homo sapiens]	44.6	23770	FKBP8	16	65.90171604	77263.43079	5091792.676	0	4
Q08378	Golgin subfamily A member 3 [OS=Homo sapiens]	167.3	2802	GOLGA3	33	63.13970458	67943.10119	4289907.338	0	4
P08670	Vimentin [OS=Homo sapiens]	53.6	7431	VIM	6	61.37850148	8082.22688	496074.9745	0	4
P04264	Keratin, type II cytoskeletal 1 [OS=Homo sapiens]	66	3848	KRT1	49	57.3574577	719822.6483	41287197.1	0	4
Q96A49	Synapse-associated protein 1 [OS=Homo sapiens]	39.9	94056	SYAP1	37	55.62948996	2019178.355	112325862	0	4
P41236	Protein phosphatase inhibitor 2 [OS=Homo sapiens]	23	5504	PPP1R2	4	53.47338072	13664.89602	730708.1875	0	4
A0FGR8-6	Isoform 6 of Extended synaptotagmin-2 [OS=Homo sapiens]	104.6	57488	ESYT2	10	53.23119276	25492.93351	1357019.258	0	4
P35527	Keratin, type I cytoskeletal 9 [OS=Homo sapiens]	62	3857	KRT9	31	52.56936292	575130.5332	30234245.73	0	4
Q9H3P7	Golgi resident protein GCP60 [OS=Homo sapiens]	60.6	64746	ACBD3	3	52.4266199	7541.415917	395370.9458	0	4
P21333	Filamin-A [OS=Homo sapiens]	280.6	2316	FLNA	29	49.29714819	83635.37057	4122985.257	0	4
Q9Y4P8-1	WD repeat domain phosphoinositide-interacting protein 2 [OS=Homo sapiens]	49.4	26100	WIPI2	2	49.15513937	2203.283593	108302.7121	0	3
Q9HB71	Calcyclin-binding protein [OS=Homo sapiens]	26.2	27101	CACYBP	12	48.71107856	42937.86987	2091549.952	0	4
O43175	D-3-phosphoglycerate dehydrogenase [OS=Homo sapiens]	56.6	26227	PHGDH	3	47.74403956	5321.963067	254092.0152	0	4
Q8N5G2-1	macoilin [OS=Homo sapiens]	76.1	55219	TMEM57; MACO1	18	47.11705859	63785.90417	3005404.184	0	4
P00387-3	Isoform 3 of NADH-cytochrome b5 reductase 3 [OS=Homo sapiens]	38.2	1727	CYB5R3	1	46.54521495	2469.322764	114935.1588	0	4
Q9UL25	Ras-related protein Rab-21 [OS=Homo sapiens]	24.3	23011	RAB21	4	46.17289406	32246.89906	1488932.654	0	4
Q9BZF1-1	Oxysterol-binding protein-related protein 8 [OS=Homo sapiens]	101.1	114882	OSBPL8	17	45.98241589	70355.95277	3235136.681	0	4
O14654	insulin receptor substrate 4 [OS=Homo sapiens]	133.7	8471	IRS4	2	44.77598367	9892.111948	442929.043	0	4
P55010	Eukaryotic translation initiation factor 5 [OS=Homo sapiens]	49.2	1983	EIF5	8	43.18385761	39375.47459	1700384.888	0	3
P11940-1	Polyadenylate-binding protein 1 [OS=Homo sapiens]	70.6	26986	PABPC1	2	42.6090995	6150.319612	262059.5803	0	4
Q641Q2	WASH complex subunit 2A [OS=Homo sapiens]	147.1	387680	FAM21A; WASHC2A	7	42.45095245	9070.055851	385032.5097	0	3
P39880-3	Isoform 3 of Homeobox protein cut-like 1 [OS=Homo sapiens]	165.6	1523	CUX1	14	36.34324858	65748.94796	2389530.359	0	4
Q5VT06	Centrosome-associated protein 350 [OS=Homo sapiens]	350.7	9857	CEP350	10	35.52306544	24050.54908	854349.229	0	4
O95551-2	Isoform 2 of Tyrosyl-DNA phosphodiesterase 2 [OS=Homo sapiens]	44.3	51567	TDP2	1	31.99716726	1917.697813	61360.89767	0	4
P20700	Lamin-B1 [OS=Homo sapiens]	66.4	4001	LMNB1	8	30.9558793	20452.07842	633112.0711	0	4
Q09666-1	Neuroblast differentiation-associated protein AHNAK [OS=Homo sapiens]	628.7	79026	AHNAK	38	27.00541053	133753.7615	3612075.238	0	4
Q96SK2-2	Isoform 2 of Transmembrane protein 209 [OS=Homo sapiens]	62.8	84928	TMEM209	7	26.75221735	26430.77304	707081.785	0	4
Q9H6H4-1	Receptor expression-enhancing protein 4 [OS=Homo sapiens]	29.4	80346	REEP4	2	26.61073959	6877.73518	183021.6198	0	4
Q96C19	EF-hand domain-containing protein D2 [OS=Homo sapiens]	26.7	79180	EFHD2	5	26.30564573	50115.6225	1318323.811	0	4
Q9H2M9	Rab3 GTPase-activating protein non-catalytic subunit [OS=Homo sapiens]	155.9	25782	RAB3GAP2	13	25.35536893	78662.87733	1994526.276	0	4
Q14789	Golgin subfamily B member 1 [OS=Homo sapiens]	375.8	2804	GOLGB1	65	25.02236249	322305.7482	8064851.264	0	2
MuV_Odate_M	MuV_Odate_M protein	41.6			14	24.06946165	308892.9003	7434885.82	0	4
Q15042-3	Isoform 2 of Rab3 GTPase-activating protein catalytic subunit [OS=Homo sapiens]	111.2	22930	RAB3GAP1	9	24.02605876	39738.05087	954748.7453	0	4
Q14739	Lamin-B receptor [OS=Homo sapiens]	70.7	3930	LBR	21	23.03966887	4172877.329	96141711.89	0	3
Q15366-2	Isoform 2 of Poly(rC)-binding protein 2 [OS=Homo sapiens]	38.6	5094	PCBP2	4	21.9284346	41866.84252	918074.318	0	4
P55072	Transitional endoplasmic reticulum ATPase [OS=Homo sapiens]	89.3	7415	VCP	2	18.72303012	3754.62296	70297.91875	0	4
Q9UGI8	Testin [OS=Homo sapiens]	48	26136	TES	6	18.09062693	72368.85825	1309198.016	0	2
Q9UGP8	Translocation protein SEC63 homolog [OS=Homo sapiens]	87.9	11231	SEC63	28	17.99207907	769145.4721	13838526.15	0	4
Q9Y6X4-1	Soluble lamin-associated protein of 75 kDa [OS=Homo sapiens]	74.9	26049	FAM169A	3	16.78012044	29220.49247	490323.3828	0	2
P11021	78 kDa glucose-regulated protein [OS=Homo sapiens]	72.3	3309	HSPA5	10	16.55579606	47858.9093	792342.3421	0	4
Q6SZW1	Sterile alpha and TIR motif-containing protein 1 [OS=Homo sapiens]	79.3	23098	SARM1	2	15.35314886	99733.31472	1531220.428	0	4
P11586	C-1-tetrahydrofolate synthase, cytoplasmic [OS=Homo sapiens]	101.5	4522	MTHFD1	3	15.33514394	14793.82136	226865.38	0	4
Q8WVM8	Sec1 family domain-containing protein 1 [OS=Homo sapiens]	72.3	23256	SCFD1	13	15.19506521	186359.1456	2831739.371	0	4
Q96KM6	zinc finger protein 512B [OS=Homo sapiens]	97.2	57473	ZNF512B	2	14.74188113	212650.9145	3134874.505	0	3
Q96QD8	sodium-coupled neutral amino acid transporter 2 [OS=Homo sapiens]	56	54407	SLC38A2	2	14.36394499	10812.66154	155312.4755	0	1
Q13439-5	Isoform 5 of Golgin subfamily A member 4 [OS=Homo sapiens]	262.2	2803	GOLGA4	26	12.59651294	162594.9694	2048129.636	0	4
Q8WYP5-2	Isoform 2 of Protein ELYS [OS=Homo sapiens]	255.8	25909	AHCTF1	28	11.47725752	358595.3176	4115690.804	0	1
P08238	Heat shock protein HSP 90-beta [OS=Homo sapiens]	83.2	3326	HSP90AB1	20	11.02175016	314437.1596	3465647.815	0	4
P13645	Keratin, type I cytoskeletal 10 [OS=Homo sapiens]	58.8	3858	KRT10	11	10.45203006	200487.8915	2095505.469	0	2
P22061-2	Isoform 2 of Protein-L-isoaspartate(D-aspartate) O-methyltransferase [OS=Homo sapiens]	24.7	5110	PCMT1	2	10.19215225	9227.938453	94052.55365	0	2
O60271-2	Isoform 2 of C-Jun-amino-terminal kinase-interacting protein 4 [OS=Homo sapiens]	145	9043	SPAG9	28	9.916783975	812333.2469	8055733.325	0	3
P49257	Protein ERGIC-53 [OS=Homo sapiens]	57.5	3998	LMAN1	12	8.550529161	193906.0416	1657999.263	0	4
Q9P0L0	vesicle-associated membrane protein-associated protein A [OS=Homo sapiens]	27.9	9218	VAPA	12		0	48747201.62	4	4
O95292	Vesicle-associated membrane protein-associated protein B/C [OS=Homo sapiens]	27.2	9217	VAPB	21		0	36786843.2	4	4
Q8N5K1	CDGSH iron-sulfur domain-containing protein 2 [OS=Homo sapiens]	15.3	493856	CISD2	6		0	7682509.302	2	4
O75396	Vesicle-trafficking protein SEC22b [OS=Homo sapiens]	24.6	9554	SEC22B	13		0	10474423.49	4	4

Q07065	Cytoskeleton-associated protein 4 [OS=Homo sapiens]	66	10970	CKAP4	43	0	25726505.8	4	4
Q5JTV8-3	Isoform 3 of Torsin-1A-interacting protein 1 [OS=Homo sapiens]	66.3	26092	TOR1AIP1	25	0	20511307.09	3	4
P37840-1	Alpha-synuclein [OS=Homo sapiens]	14.5	6622	SNCA	8	0	4238145.031	0	4
Q96HY6-1	DDRGK domain-containing protein 1 [OS=Homo sapiens]	35.6	65992	DDRGK1	3	0	8632799.795	4	4
Q96S66	chloride channel CLIC-like protein 1 [OS=Homo sapiens]	62	23155	CLCC1	14	0	13523561.96	0	4
Q9H910-3	Isoform 3 of Jupiter microtubule associated homolog 2 [OS=Homo sapiens]	23	90861	HN1L; JPT2	13	0	4656025.133	1	4
P84157-1	Matrix-remodeling-associated protein 7 [OS=Homo sapiens]	21.5	439921	MXRA7	10	0	4349019.713	4	4
O95372	acyl-protein thioesterase 2 [OS=Homo sapiens]	24.7	11313	LYPLA2	5	0	4824651.322	1	4
P84157-2	Isoform 2 of Matrix-remodeling-associated protein 7 [OS=Homo sapiens]	17.5	439921	MXRA7	6	0	3505862.258	2	4
O43768-4	Isoform 4 of Alpha-endosulfine [OS=Homo sapiens]	15.5	2029	ENSA	6	0	2817346.385	4	4
Q12981	Vesicle transport protein SEC20 [OS=Homo sapiens]	26.1	662	BNIP1	14	0	3337543.803	1	4
Q9H444	Charged multivesicular body protein 4b [OS=Homo sapiens]	24.9	128866	CHMP4B	7	0	3209259.702	4	4
Q13177	Serine/threonine-protein kinase PAK 2 [OS=Homo sapiens]	58	5062	PAK2	20	0	7350714.908	4	4
Q04760-1	lactoylglutathione lyase [OS=Homo sapiens]	20.8	2739	GLO1	11	0	2357506.15	2	4
Q99442	translocation protein sec62 [OS=Homo sapiens]	45.8	7095	SEC62	4	0	4657372.932	1	4
Q5HYI8	Rab-like protein 3 [OS=Homo sapiens]	26.4	285282	RABL3	3	0	2698477.911	4	4
P61956	small ubiquitin-related modifier 2 [OS=Homo sapiens]	10.9	6613	SUMO2	2	0	1076534.913	4	4
Q9UHB9	Signal recognition particle subunit SRP68 [OS=Homo sapiens]	70.7	6730	SRP68	31	0	6945193.444	4	4
Q9Y6X1	Stress-associated endoplasmic reticulum protein 1 [OS=Homo sapiens]	7.4	27230	SERP1	2	0	723678.7593	0	4
O75410-2	Isoform 2 of Transforming acidic coiled-coil-containing protein 1 [OS=Homo sapiens]	89.2	6867	TACC1	25	0	8746587.414	2	4
Q8NHP6-1	motile sperm domain-containing protein 2 [OS=Homo sapiens]	59.7	158747	MOSPD2	11	0	5308190.643	0	4
Q9BVC6	Transmembrane protein 109 [OS=Homo sapiens]	26.2	79073	TMEM109	3	0	2186730.775	4	4
P52594-4	Isoform 4 of Arf-GAP domain and FG repeat-containing protein 1 [OS=Homo sapiens]	60.7	3267	AGFG1	9	0	5155594.782	3	4
Q8WW12	PEST proteolytic signal-containing nuclear protein [OS=Homo sapiens]	18.9	57092	PCNP	4	0	1529091.816	0	4
Q9Y679-1	Ancient ubiquitous protein 1 [OS=Homo sapiens]	53	550	AUP1	10	0	3842869.714	2	4
Q9Y5M8	signal recognition particle receptor subunit beta [OS=Homo sapiens]	29.7	58477	SRPRB	3	0	2086510.529	2	4
Q9UHD1	cysteine and histidine-rich domain-containing protein 1 [OS=Homo sapiens]	37.5	26973	CHORDC1	5	0	2433566.727	1	4
Q14677-3	Isoform 3 of Clathrin interactor 1 [OS=Homo sapiens]	70.3	9685	CLINT1	12	0	4560511.579	1	4
P43307	Translocon-associated protein subunit alpha [OS=Homo sapiens]	32.2	6745	SSR1	2	0	1945545.782	3	4
Q9UKY7	Protein CDV3 homolog [OS=Homo sapiens]	27.3	55573	CDV3	7	0	1615517.52	4	4
O00193	Small acidic protein [OS=Homo sapiens]	20.3	10944	C11orf58	3	0	1130606.067	3	4
P42167-2	Isoform Gamma of Lamina-associated polypeptide 2, isoforms beta/gamma [OS=Homo sapiens]	38.7	7112	TMPO	35	0	2126794.605	0	4
P48444	Coatomer subunit delta [OS=Homo sapiens]	57.2	372	ARCN1	11	0	3096534.412	4	4
P62820	Ras-related protein Rab-1A [OS=Homo sapiens]	22.7	5861	RAB1A	7	0	1188000.878	4	4
Q15691	Microtubule-associated protein RP/EB family member 1 [OS=Homo sapiens]	30	22919	MAPRE1	6	0	1489651.743	1	4
Q7Z3B0	Small integral membrane protein 15 [OS=Homo sapiens]	8.6	643155	C5orf43; SMIM15	1	0	397105.2089	3	4
Q00765	Receptor expression-enhancing protein 5 [OS=Homo sapiens]	21.5	7905	REEP5	3	0	1006902.937	3	4
Q9C0E8-4	Isoform 4 of Protein lunapark [OS=Homo sapiens]	50.8	80856	KIAA1715; LNPK	8	0	2423605.026	0	4
Q9UKK9	ADP-sugar pyrophosphatase [OS=Homo sapiens]	24.3	11164	NUDT5	6	0	1120417.252	0	4
P61923	Coatomer subunit zeta-1 [OS=Homo sapiens]	20.2	22818	COPZ1	5	0	864649.9384	2	4
Q15382	GTP-binding protein rheb [OS=Homo sapiens]	20.5	6009	RHEB	6	0	847243.56	0	4
P35610	sterol O-acyltransferase 1 [OS=Homo sapiens]	64.7	6646	SOAT1	3	0	2404185.966	1	4
Q96JB5	CDK5 regulatory subunit-associated protein 3 [OS=Homo sapiens]	56.9	80279	CDK5RAP3	13	0	2203999.779	1	4
Q9Y385	ubiquitin-conjugating enzyme e2 j1 [OS=Homo sapiens]	35.2	51465	UBE2J1	2	0	1371576.039	0	4
Q8TBA6	Golgin subfamily A member 5 [OS=Homo sapiens]	83	9950	GOLGA5	18	0	3113157.693	0	4
Q8N4V1-2	Isoform 2 of Membrane magnesium transporter 1 [OS=Homo sapiens]	21.9	93380	MMGT1	4	0	829209.9426	0	4
P14635	G2/mitotic-specific cyclin-B1 [OS=Homo sapiens]	48.3	891	CCNB1	7	0	1821393.271	0	4
P78318	Immunoglobulin-binding protein 1 [OS=Homo sapiens]	39.2	3476	IGBP1	6	0	1349442.527	4	4
Q9H8Y8-3	Isoform 3 of Golgi reassembly-stacking protein 2 [OS=Homo sapiens]	48.6	26003	GORASP2	5	0	1827141.69	0	4
Q96F86	Enhancer of mRNA-decapping protein 3 [OS=Homo sapiens]	56	80153	EDC3	5	0	1984171.964	4	4
Q99618	Cell division cycle-associated protein 3 [OS=Homo sapiens]	29	83461	CDCA3	7	0	1022767.481	1	4
Q07960	rho GTPase-activating protein 1 [OS=Homo sapiens]	50.4	392	ARHGAP1	12	0	1662539.53	4	4
Q8N6T3-1	ADP-ribosylation factor GTPase-activating protein 1 [OS=Homo sapiens]	44.6	55738	ARFGAP1	8	0	1511884.707	4	4
Q99733-2	Isoform 2 of Nucleosome assembly protein 1-like 4 [OS=Homo sapiens]	44.1	4676	NAP1L4	4	0	1432104.322	4	4
Q86UL3	glycerol-3-phosphate acyltransferase 4 [OS=Homo sapiens]	52	137964	AGPAT6; GPAT4	6	0	1671775.465	1	4
O14737	Programmed cell death protein 5 [OS=Homo sapiens]	14.3	9141	PDCD5	5	0	446395.2034	2	4
P46060	Ran GTPase-activating protein 1 [OS=Homo sapiens]	63.5	5905	RANGAP1	12	0	2002128.238	4	4
O43399	Tumor protein D54 [OS=Homo sapiens]	22.2	7165	TPD52L2	4	0	699907.4343	0	4
P61758	Prefoldin subunit 3 [OS=Homo sapiens]	22.6	7411	VBP1	4	0	621868.7109	0	4
Q6IAA8	ragulator complex protein LAMTOR1 [OS=Homo sapiens]	17.7	55004	LAMTOR1	2	0	506600.0497	0	4
O43765	Small glutamine-rich tetratricopeptide repeat-containing protein alpha [OS=Homo sapiens]	34	6449	SGTA	2	0	976575.8941	0	4
Q9BQE4	selenoprotein S [OS=Homo sapiens]	21.2	55829	VIMP; SELENOS	3	0	561906.0938	2	4
Q9NYM9	BET1-like protein [OS=Homo sapiens]	12.4	51272	BET1L	3	0	313875.2097	4	4
P05387	60S acidic ribosomal protein P2 [OS=Homo sapiens]	11.7	6181	RPLP2	3	0	328469.9416	4	4
Q16143	Beta-synuclein [OS=Homo sapiens]	14.3	6620	SNCB	4	0	378033.1665	0	4
P02533	Keratin, type I cytoskeletal 14 [OS=Homo sapiens]	51.5	3861	KRT14	11	0	1314558.143	3	4
Q9Y5X3	sorting nexin-5 [OS=Homo sapiens]	46.8	27131	SNX5	9	0	1103917.068	0	4
Q99961	Endophilin-A2 [OS=Homo sapiens]	41.5	6455	SH3GL1	5	0	998295.7543	0	4
Q99614	tetratricopeptide repeat protein 1 [OS=Homo sapiens]	33.5	7265	TTC1	4	0	776987.4597	0	4
P05556-1	Integrin beta-1 [OS=Homo sapiens]	88.4	3688	ITGB1	11	0	2123793.619	0	4
Q6ZVM7-5	Isoform 5 of TOM1-like protein 2 [OS=Homo sapiens]	53.3	146691	TOM1L2	1	0	1276014.818	0	4
O95210	Starch-binding domain-containing protein 1 [OS=Homo sapiens]	39	8987	STBD1	4	0	934925.9927	1	4
Q16543	Hsp90 co-chaperone Cdc37 [OS=Homo sapiens]	44.4	11140	CDC37	7	0	972049.6173	0	4
Q15643	Thyroid receptor-interacting protein 11 [OS=Homo sapiens]	227.4	9321	TRIP11	37	0	5037225.605	0	4
P63165-1	Small ubiquitin-related modifier 1 [OS=Homo sapiens]	11.6	7341	SUMO1	1	0	248845.6105	3	4
P04843	Dolichyl-diphosphooligosaccharide--protein glycosyltransferase subunit 1 [OS=Homo sapiens]	68.5	6184	RPN1	10	0	1347109.523	3	4
Q9Y6I9	Testis-expressed protein 264 [OS=Homo sapiens]	34.2	51368	TEX264	4	0	687475.8465	3	4
O95801	Tetratricopeptide repeat protein 4 [OS=Homo sapiens]	44.7	7268	TTC4	4	0	849051.0032	0	4
Q9NXW2-2	Isoform 2 of DnaJ homolog subfamily B member 12 [OS=Homo sapiens]	42.2	54788	DNAJB12	7	0	820164.8742	2	4
Q629K1	Triple QxxK/R motif-containing protein [OS=Homo sapiens]	9.7	286144	TRIQK	1	0	176881.0725	0	4
Q9UK76-2	Isoform 2 of Jupiter microtubule associated homolog 1 [OS=Homo sapiens]	19.9	51155	HN1; JPT1	2	0	369026.6199	0	4
Q6JEL2	Kelch-like protein 10 [OS=Homo sapiens]	68.9	317719	KLHL10	1	0	1241863.873	2	4
Q5JSH3	WD repeat-containing protein 44 [OS=Homo sapiens]	101.3	54521	WDR44	16	0	1842328.423	1	4
P00167-1	Cytochrome b5 [OS=Homo sapiens]	15.3	1528	CYB5A	2	0	270640.2277	0	4
Q9BTX1	Nucleoporin Ndc1 [OS=Homo sapiens]	76.3	55706	NDC1; TMEM48	9	0	1356565.169	4	4
P23280-2	Isoform 2 of Carbonic anhydrase 6 [OS=Homo sapiens]	35.3	765	CA6	1	0	440960.7861	4	4
Q04637-9	Isoform 9 of Eukaryotic translation initiation factor 4 gamma 1 [OS=Homo sapiens]	176.1	1981	EIF4G1	11	0	2841138.613	3	4
Q6P996-1	Pyridoxal-dependent decarboxylase domain-containing protein 1 [OS=Homo sapiens]	86.7	23042; 102724985	PDXDC1; LOC102724985	9	0	1296549.958	1	4
Q6P1N0	Coiled-coil and C2 domain-containing protein 1A [OS=Homo sapiens]	104	54862	CC2D1A	9	0	1528701.573	2	4
O95573	long-chain-fatty-acid--CoA ligase 3 [OS=Homo sapiens]	80.4	2181	ACSL3	8	0	1102596.071	0	4
Q9UJU6-2	Isoform 2 of Drebrin-like protein [OS=Homo sapiens]	48.3	28988	DBNL	5	0	658522.9141	1	4
P63027	Vesicle-associated membrane protein 2 [OS=Homo sapiens]	12.7	6844	VAMP2	1	0	174938.5348	0	4
Q86SQ9-4	Isoform 4 of Dehydrodolichyl diphosphate synthase complex subunit DHDDS [OS=Homo sapiens]	34.8	79947	DHDDS	1	0	448425.1984	0	4
O15126-1	Secretory carrier-associated membrane protein 1 [OS=Homo sapiens]	37.9	9522	SCAMP1	4	0	504371.2988	1	4
Q9NZT1	Calmodulin-like protein 5 [OS=Homo sapiens]	15.9	51806	CALML5	1	0	108084.3332	0	4
Q15942	Zyxin [OS=Homo sapiens]	61.2	7791	ZYX	6	0	838948.1428	1	4
P49792	E3 SUMO-protein ligase RanBP2 [OS=Homo sapiens]	358	5903	RANBP2	36	0	4615021.77	1	4
P16333-1	Cytoplasmic protein NCK1 [OS=Homo sapiens]	42.8	4690	NCK1	4	0	525432.4855	1	4
Q9NZZ3	Charged multivesicular body protein 5 [OS=Homo sapiens]	24.6	51510	CHMP5	3	0	300256.9739	0	4
O00124-1	UBX domain-containing protein 8 [OS=Homo sapiens]	30.5	7993	UBXN8	3	0	363147.4771	4	4
Q9Y320	Thioredoxin-related transmembrane protein 2 [OS=Homo sapiens]	34	51075	TMX2	4	0	384313.0498	0	4
O00161	Synaptosomal-associated protein 23 [OS=Homo sapiens]	23.3	8773	SNAP23	4	0	273312.352	3	4
Q9H6T3	RNA polymerase II-associated protein 3 [OS=Homo sapiens]	75.7	79657	RPAP3	12	0	854226.389	4	4
O15269	serine palmitoyltransferase 1 [OS=Homo sapiens]	52.7	10558	SPTLC1	3	0	600389.4247	0	4
Q13426-1	DNA repair protein xrcc4 [OS=Homo sapiens]	38.3	7518	XRCC4	1	0	419370.3401	1	4
O14653-2	Isoform B of Golgi SNAP receptor complex member 2 [OS=Homo sapiens]	24.6	9570	GOSR2	1	0	259693.948	0	4
Q7Z3T8	Zinc finger FYVE domain-containing protein 16 [OS=Homo sapiens]	168.8	9765	ZFYVE16	11	0	1867531.733	2	4
P55209	Nucleosome assembly protein 1-like 1 [OS=Homo sapiens]	45.3	4673	NAP1L1	4	0	464483.4602	0	4
Q15738	sterol-4-alpha-carboxylate 3-dehydrogenase, decarboxylating [OS=Homo sapiens]	41.9	50814	NSDHL	5	0	440466.7698	0	4
Q8TEV9	Guanine nucleotide exchange protein SMCR8 [OS=Homo sapiens]	105	140775	SMCR8	8	0	1103779.543	0	4
A0MZ66-3	Isoform 3 of Shootin-1 [OS=Homo sapiens]	73.6	57698	KIAA1598; SHTN1	9	0	753143.6668	2	4
Q5TFG8	zinc finger C2HC domain-containing protein 1B [OS=Homo sapiens]	24.7	153918	ZC2HC1B	1	0	255848.6593	0	4
Q8N2K1-3	Isoform 3 of Ubiquitin-conjugating enzyme E2 J2 [OS=Homo sapiens]	31	118424	UBE2J2	1	0	82928.9317	0	4
O75822	Eukaryotic translation initiation factor 3 subunit J [OS=Homo sapiens]	29	8669	EIF3J	2	0	294172.9328	0	4
Q8N5M4	Tetratricopeptide repeat protein 9C [OS=Homo sapiens]	20	283237	TTC9C	2	0	194410.9654	0	4
Q9NVZ3-2	Isoform 2 of Adaptin ear-binding coat-associated protein 2 [OS=Homo sapiens]	29.4	55707	NECAP2	2	0	296317.3889	0	4
Q96N66-1	Lysophospholipid acyltransferase 7 [OS=Homo sapiens]	52.7	79143	MBOAT7	1	0	510570.2611	1	4
P78310-1	Coxsackievirus and adenovirus receptor [OS=Homo sapiens]	40	1525	CXADR	5	0	394497.9868	4	4
Q8TC12	Retinol dehydrogenase 11 [OS=Homo sapiens]	35.4	51109	RDH11	4	0	331156.7178	1	4
Q9HCU5	Prolactin regulatory element-binding protein [OS=Homo sapiens]	45.4	10113	PREB	4	0	433074.6401	0	4
O14668-1	Transmembrane gamma-carboxyglutamic acid protein 1 [OS=Homo sapiens]	24.9	5638	PRRG1	1	0	216506.1367	0	4
Q8NEN9	PDZ domain-containing protein 8 [OS=Homo sapiens]	128.5	118987	PDZD8	6	0	1142789.113	4	4
O75575-1	DNA-directed RNA polymerase III subunit RPC9 [OS=Homo sapiens]	16.9	27297	CRCP	2	0	142655.6014	4	4
Q9Y2Z0-1	Protein SGT1 homolog [OS=Homo sapiens]	41	10910	SUGT1	4	0	350454.4347	1	4
Q96HE9	Proline-rich protein 11 [OS=Homo sapiens]	40.1	55771	PRR11	3	0	341618.4979	3	4
Q9NP61-1	ADP-ribosylation factor GTPase-activating protein 3 [OS=Homo sapiens]	56.9	26286	ARFGAP3	4	0	489691.8226	4	4
Q9NQC3	Reticulon-4 [OS=Homo sapiens]	129.9	57142	RTN4	10	0	1125960.723	0	4
Q96AC1-3	Isoform 3 of Fermitin family homolog 2 [OS=Homo sapiens]	78.6	10979	FERMT2	8	0	630548.3	0	4
P50851-2	Isoform 2 of Lipopolysaccharide-responsive and beige-like anchor protein [OS=Homo sapiens]	317.5	987	LRBA	18	0	2592613.194	0	4
Q9UHB7-1	AF4/FMR2 family member 4 [OS=Homo sapiens]	127.4	27125	AFF4	1	0	1050510.706	0	4
Q6P1M0	Long-chain fatty acid transport protein 4 [OS=Homo sapiens]	72	10999	SLC27A4	4	0	572832.959	1	4
O14810	Complexin-1 [OS=Homo sapiens]	15	10815	CPLX1	2	0	119074.2951	0	4
O60493-1	sorting nexin-3 [OS=Homo sapiens]	18.8	8724	SNX3	3	0	139250.5881	0	4
Q9ULV4-3	Isoform 3 of Coronin-1C [OS=Homo sapiens]	58.9	23603	CORO1C	5	0	447555.3174	3	4
Q96EA4	protein Spindly [OS=Homo sapiens]	70.1	54908	SPDL1	8	0	497327.4459	0	4
O95793	double-stranded RNA-binding protein Staufen homolog 1 [OS=Homo sapiens]	63.1	6780	STAU1	5	0	472812.9822	3	4
P78536-1	Disintegrin and metalloproteinase domain-containing protein 17 [OS=Homo sapiens]	93	6868	ADAM17	5	0	650826.4802	0	4
Q9UKX7	Nuclear pore complex protein Nup50 [OS=Homo sapiens]	50.1	10762	NUP50	3	0	365845.2795	0	4
P53367	Arfaptin-1 [OS=Homo sapiens]	41.7	27236	ARFIP1	2	0	289432.4378	0	4
O60664-1	Perilipin-3 [OS=Homo sapiens]	47	10226	PLIN3	3	0	335095.4561	0	4
P41162	ETS translocation variant 3 [OS=Homo sapiens]	57	2117	ETV3	1	0	389938.3144	0	4
O00264-2	Isoform 2 of Membrane-associated progesterone receptor component 1 [OS=Homo sapiens]	15.9	10857	PGRMC1	4	0	108161.3507	0	4
Q00013-1	55 kDa erythrocyte membrane protein [OS=Homo sapiens]	52.3	4354	MPP1	5	0	351005.4042	0	4
Q08752	peptidyl-prolyl cis-trans isomerase D [OS=Homo sapiens]	40.7	5481	PPID	3	0	277425.5357	4	4
P61006	Ras-related protein Rab-8A [OS=Homo sapiens]	23.7	4218	RAB8A	2	0	155186.2637	0	4
Q9UBM7	7-dehydrocholesterol reductase [OS=Homo sapiens]	54.5	1717	DHCR7	1	0	353705.6638	4	4
Q8TEY7-1	Ubiquitin carboxyl-terminal hydrolase 33 [OS=Homo sapiens]	106.7	23032	USP33	4	0	686706.0919	4	4
Q9HD26-1	Golgi-associated PDZ and coiled-coil motif-containing protein [OS=Homo sapiens]	50.5	57120	GOPC	2	0	325639.0549	0	4
Q32MZ4	Leucine-rich repeat flightless-interacting protein 1 [OS=Homo sapiens]	89.2	9208	LRRFIP1	5	0	553247.064	1	4
Q15843	NEDD8 [OS=Homo sapiens]	9.1	4738	NEDD8	1	0	54639.59244	4	4
Q15005	Signal peptidase complex subunit 2 [OS=Homo sapiens]	25	9789	SPCS2	1	0	154085.0445	1	4
Q02952-1	A-kinase anchor protein 12 [OS=Homo sapiens]	191.4	9590	AKAP12	10	0	1205963.806	1	4
Q9ULC3	Ras-related protein Rab-23 [OS=Homo sapiens]	26.6	51715	RAB23	3	0	159710.113	0	4
Q86TJ2-1	Transcriptional adapter 2-beta [OS=Homo sapiens]	48.4	93624	TADA2B	1	0	142255.8171	2	4
Q8NE71-1	ATP-binding cassette sub-family F member 1 [OS=Homo sapiens]	95.9	23	ABCF1	4	0	560113.7604	0	4
Q9Y282-3	Isoform 3 of Endoplasmic reticulum-Golgi intermediate compartment protein 3 [OS=Homo sapiens]	43.7	51614	ERGIC3	2	0	255010.9457	4	4
P13647	keratin, type II cytoskeletal 5 [OS=Homo sapiens]	62.3	3852	KRT5	12	0	385419.9092	1	4
P23528	Cofilin-1 [OS=Homo sapiens]	18.5	1072	CFL1	1	0	54961.0266	0	4
Q92545	Transmembrane protein 131 [OS=Homo sapiens]	205	23505	TMEM131	10	0	1208927.349	0	4
Q8TCJ2	Dolichyl-diphosphooligosaccharide--protein glycosyltransferase subunit STT3B [OS=Homo sapiens]	93.6	201595	STT3B	5	0	512494.3903	0	4
O43865	S-adenosylhomocysteine hydrolase-like protein 1 [OS=Homo sapiens]	58.9	10768	AHCYL1	5	0	326897.5226	3	4
P30101	Protein disulfide-isomerase A3 [OS=Homo sapiens]	56.7	2923	PDIA3	3	0	308202.4934	1	4
P11233	Ras-related protein Ral-A [OS=Homo sapiens]	23.6	5898	RALA	2	0	125748.7288	0	4
P17706-1	Tyrosine-protein phosphatase non-receptor type 2 [OS=Homo sapiens]	48.4	5771	PTPN2	2	0	253725.8617	2	4
Q8TBM8	dnaJ homolog subfamily B member 14 [OS=Homo sapiens]	42.5	79982	DNAJB14	1	0	223693.2582	1	4
Q6NXT6	Transmembrane anterior posterior transformation protein 1 homolog [OS=Homo sapiens]	64.2	202018	TAPT1	2	0	329743.7469	1	4
Q4KMQ2-2	Isoform 2 of Anoctamin-6 [OS=Homo sapiens]	108.4	196527	ANO6	4	0	530430.0354	3	4

Q8WUH6	Transmembrane protein 263 [OS=Homo sapiens]	11.7	90488	C12orf23; TMEM263	1	0	65183.5242	0	4
Q92597	Protein NDRG1 [OS=Homo sapiens]	42.8	10397	NDRG1	3	0	221997.3706	0	4
O00213	amyloid-beta A4 precursor protein-binding family B member 1 [OS=Homo sapiens]	77.2	322	APBB1	3	0	397094.4191	0	4
Q9UHI5-1	Large neutral amino acids transporter small subunit 2 [OS=Homo sapiens]	58.3	23428	SLC7A8	1	0	221284.5151	0	4
P22234-2	Isoform 2 of Multifunctional protein ADE2 [OS=Homo sapiens]	47.9	10606	PAICS	2	0	236498.0838	0	4
Q9Y570-4	Isoform 4 of Protein phosphatase methylesterase 1 [OS=Homo sapiens]	43.8	51400	PPME1	4	0	218726.313	0	4
P47756-2	Isoform 2 of F-actin-capping protein subunit beta [OS=Homo sapiens]	30.6	832	CAPZB	3	0	146925.1201	0	4
Q9NV66	S-adenosyl-L-methionine-dependent tRNA 4-demethylwyosine synthase [OS=Homo sapiens]	83.6	55253	TYW1	4	0	390527.798	0	4
P27482	Calmodulin-like protein 3 [OS=Homo sapiens]	16.9	810	CALML3	1	0	78768.92856	0	4
Q9HC38	Glyoxalase domain-containing protein 4 [OS=Homo sapiens]	34.8	51031	GLOD4	3	0	165599.7298	1	4
Q16799-1	Reticulon-1 [OS=Homo sapiens]	83.6	6252	RTN1	3	0	406750.3324	0	4
Q9BY44-1	Eukaryotic translation initiation factor 2A [OS=Homo sapiens]	64.9	83939	EIF2A	5	0	299453.8756	0	4
O15504	nucleoporin-like protein 2 [OS=Homo sapiens]	44.8	11097	NUPL2	2	0	214121.4663	0	4
Q8TCG1	Protein CIP2A [OS=Homo sapiens]	102.1	57650	KIAA1524; CIP2A	2	0	445316.021	1	4
O95487-3	Isoform 3 of Protein transport protein Sec24B [OS=Homo sapiens]	140.3	10427	SEC24B	4	0	636162.6062	0	4
O43491-1	band 4.1-like protein 2 [OS=Homo sapiens]	112.5	2037	EPB41L2	4	0	486584.3069	4	4
P52565-1	rho GDP-dissociation inhibitor 1 [OS=Homo sapiens]	23.2	396	ARHGDIA	2	0	99721.96939	0	4
P57737-3	Isoform 3 of Coronin-7 [OS=Homo sapiens]	114.1	100529144; 79585	CORO7-PAM16; CORO7	4	0	497718.5309	0	4
Q13596	Sorting nexin-1 [OS=Homo sapiens]	59	6642	SNX1	3	0	245143.945	0	4
Q9NYL9	tropomodulin-3 [OS=Homo sapiens]	39.6	29766	TMOD3	4	0	164379.3744	0	4
Q9UHV9	Prefoldin subunit 2 [OS=Homo sapiens]	16.6	5202	PFDN2	1	0	69202.84321	0	4
O75838-2	Isoform 2 of Calcium and integrin-binding family member 2 [OS=Homo sapiens]	24.1	10518	CIB2	1	0	93405.44692	3	4
Q9BRP8	Partner of Y14 and mago [OS=Homo sapiens]	22.6	84305	WIBG; PYM1	1	0	89620.0166	4	4
O00400	Acetyl-coenzyme A transporter 1 [OS=Homo sapiens]	60.9	9197	SLC33A1	1	0	181630.8615	0	4
Q9BXB4	Oxysterol-binding protein-related protein 11 [OS=Homo sapiens]	83.6	114885	OSBPL11	3	0	324468.8716	0	4
Q5JRA6-1	Transport and Golgi organization protein 1 homolog [OS=Homo sapiens]	213.6	375056	MIA3	9	0	825658.4537	0	4
Q96CS3	FAS-associated factor 2 [OS=Homo sapiens]	52.6	23197	FAF2	2	0	192245.4086	0	4
P55327-3	Isoform 3 of Tumor protein D52 [OS=Homo sapiens]	26.4	7163	TPD52	3	0	104910.6218	0	4
Q96E17	ras-related protein Rab-3C [OS=Homo sapiens]	25.9	115827	RAB3C	1	0	97180.55582	0	4
Q8WUX9	Charged multivesicular body protein 7 [OS=Homo sapiens]	50.9	91782	CHMP7	2	0	187695.0107	1	4
Q5SWA1	Protein phosphatase 1 regulatory subunit 15B [OS=Homo sapiens]	79.1	84919	PPP1R15B	1	0	291785.555	0	3
Q92844	TRAF family member-associated NF-kappa-B activator [OS=Homo sapiens]	47.8	10010	TANK	3	0	173639.5314	1	4
P51151	Ras-related protein Rab-9A [OS=Homo sapiens]	22.8	9367	RAB9A	1	0	82043.76573	1	4
O43237	Cytoplasmic dynein 1 light intermediate chain 2 [OS=Homo sapiens]	54.1	1783	DYNC1LI2	1	0	195244.0697	4	4
Q99615-1	DnaJ homolog subfamily C member 7 [OS=Homo sapiens]	56.4	7266	DNAJC7	3	0	193458.5929	0	4
Q96N67	Dedicator of cytokinesis protein 7 [OS=Homo sapiens]	242.4	85440	DOCK7	9	0	831535.3754	0	4
Q9Y5K6	CD2-associated protein [OS=Homo sapiens]	71.4	23607	CD2AP	4	0	240117.9801	0	4
Q70UQ0	Inhibitor of nuclear factor kappa-B kinase-interacting protein [OS=Homo sapiens]	39.3	121457	IKBIP	3	0	132812.0463	4	4
Q92734-1	Protein TFG [OS=Homo sapiens]	43.4	10342	TFG	3	0	147024.7881	4	4
Q9Y4P3	Transducin beta-like protein 2 [OS=Homo sapiens]	49.8	26608	TBL2	3	0	164221.9877	0	4
Q15056-1	Eukaryotic translation initiation factor 4H [OS=Homo sapiens]	27.4	7458	EIF4H	1	0	87170.37678	0	4
Q8N7C3	Probable E3 ubiquitin-protein ligase TRIML2 [OS=Homo sapiens]	44	205860	TRIML2	1	0	134752.4028	1	4
P09211	Glutathione S-transferase P [OS=Homo sapiens]	23.3	2950	GSTP1	1	0	73034.96655	0	4
O00571	ATP-dependent RNA helicase DDX3X [OS=Homo sapiens]	73.2	1654	DDX3X	6	0	230251.7009	2	4
O60763-2	Isoform 2 of General vesicular transport factor p115 [OS=Homo sapiens]	109.1	8615	USO1	5	0	327318.9778	0	4
Q9UH65	Switch-associated protein 70 [OS=Homo sapiens]	69	23075	SWAP70	3	0	189689.5147	0	4
O43402	ER membrane protein complex subunit 8 [OS=Homo sapiens]	23.8	10328	EMC8	2	0	68239.1747	0	2
Q6NTF9-2	Isoform 2 of Rhomboid domain-containing protein 2 [OS=Homo sapiens]	43.8	57414	RHBDD2	1	0	130705.9133	0	4
O95757	Heat shock 70 kDa protein 4L [OS=Homo sapiens]	94.5	22824	HSPA4L	3	0	265181.7298	4	4
Q9BXF6	Rab11 family-interacting protein 5 [OS=Homo sapiens]	70.4	26056	RAB11FIP5	3	0	201472.7645	3	4
Q8IYA7	Homeobox protein Mohawk [OS=Homo sapiens]	39.3	283078	MKX	1	0	104007.7034	0	4
Q8IYB5	Stromal membrane-associated protein 1 [OS=Homo sapiens]	50.4	60682	SMAP1	1	0	136615.216	1	4
Q9BSK2	Solute carrier family 25 member 33 [OS=Homo sapiens]	35.4	84275	SLC25A33	1	0	93078.2877	0	4
Q96SU4-6	Isoform 6 of Oxysterol-binding protein-related protein 9 [OS=Homo sapiens]	84.2	114883	OSBPL9	1	0	215964.4457	0	4
Q92551	Inositol hexakisphosphate kinase 1 [OS=Homo sapiens]	50.2	9807	IP6K1	2	0	126860.695	0	4
P82094	TATA element modulatory factor [OS=Homo sapiens]	122.8	7110	TMF1	6	0	313842.4972	0	4
Q9NW15-1	Anoctamin-10 [OS=Homo sapiens]	76.3	55129	ANO10	2	0	187339.7086	0	4
P35241-5	Isoform 5 of Radixin [OS=Homo sapiens]	71	5962	RDX	8	0	166799.4006	0	4
Q9Y3F4-2	Isoform 2 of Serine-threonine kinase receptor-associated protein [OS=Homo sapiens]	39.8	11171	STRAP	3	0	99708.14411	0	4
Q9H2H8-1	peptidyl-prolyl cis-trans isomerase-like 3 [OS=Homo sapiens]	18.1	53938	PPIL3	2	0	42931.44812	3	4
Q9NYG2-2	Isoform 2 of Palmitoyltransferase ZDHHC3 [OS=Homo sapiens]	37.3	51304	ZDHHC3	2	0	88430.53477	0	4
O14681	Etoposide-induced protein 2.4 homolog [OS=Homo sapiens]	38.9	9538	EI24	1	0	91161.45927	0	4
Q9NZ52	ADP-ribosylation factor-binding protein GGA3 [OS=Homo sapiens]	78.3	23163	GGA3	1	0	97510.65848	4	4
Q96MW1	Coiled-coil domain-containing protein 43 [OS=Homo sapiens]	25.2	124808	CCDC43	1	0	58649.54583	0	4
Q9BWL3	Uncharacterized protein C1orf43 [OS=Homo sapiens]	28.8	25912	C1orf43	1	0	65846.29468	0	4
Q9Y2G8	DnaJ homolog subfamily C member 16 [OS=Homo sapiens]	90.5	23341	DNAJC16	2	0	202168.1012	0	4
P33176	Kinesin-1 heavy chain [OS=Homo sapiens]	109.6	3799	KIF5B	4	0	247582.963	0	4
O43639	Cytoplasmic protein NCK2 [OS=Homo sapiens]	42.9	8440	NCK2	1	0	97614.24415	0	4
Q15011-1	homocysteine-responsive endoplasmic reticulum-resident ubiquitin-like domain member 1 protein [OS=Homo sapiens]	43.7	9709	HERPUD1	2	0	99907.73554	0	4
P24534	Elongation factor 1-beta [OS=Homo sapiens]	24.7	1933	EEF1B2	2	0	56768.14995	0	1
Q14BN4	Sarcolemmal membrane-associated protein [OS=Homo sapiens]	95.1	7871	SLMAP	2	0	209111.9725	0	4
Q96JQ2	Calmin [OS=Homo sapiens]	111.6	79789	CLMN	1	0	247555.2717	0	4
Q14157-5	Isoform 5 of Ubiquitin-associated protein 2-like [OS=Homo sapiens]	116.6	9898	UBAP2L	5	0	272018.2314	0	4
P35658-5	Isoform 5 of Nuclear pore complex protein Nup214 [OS=Homo sapiens]	215.3	8021	NUP214	3	0	506896.6104	0	4
O75821	Eukaryotic translation initiation factor 3 subunit G [OS=Homo sapiens]	35.6	8666	EIF3G	1	0	77676.41856	0	4
P56856-1	Claudin-18 [OS=Homo sapiens]	27.8	51208	CLDN18	1	0	30470.98066	3	4
P53621-2	Isoform 2 of Coatomer subunit alpha [OS=Homo sapiens]	139.2	1314	COPA	5	0	296288.8097	4	4
O14523-2	Isoform 2 of Phospholipid transfer protein C2CD2L [OS=Homo sapiens]	76.1	9854	C2CD2L	2	0	165939.1567	0	4
Q9BQE5	apolipoprotein L2 [OS=Homo sapiens]	37.1	23780	APOL2	1	0	78814.96788	3	4
P51648-2	Isoform 2 of Fatty aldehyde dehydrogenase [OS=Homo sapiens]	57.6	224	ALDH3A2	3	0	119338.6942	0	4
Q96H20-1	Vacuolar-sorting protein SNF8 [OS=Homo sapiens]	28.8	11267	SNF8	1	0	59400.17159	1	4
Q14258	E3 ubiquitin/ISG15 ligase TRIM25 [OS=Homo sapiens]	70.9	7706	TRIM25	2	0	138380.9673	0	4
A1X283	SH3 and PX domain-containing protein 2B [OS=Homo sapiens]	101.5	285590	SH3PXD2B	1	0	196222.6685	0	4
P26639-2	Isoform 2 of Threonine--tRNA ligase, cytoplasmic [OS=Homo sapiens]	86.8	6897	TARS	1	0	161407.2952	1	4
Q13492-1	Phosphatidylinositol-binding clathrin assembly protein [OS=Homo sapiens]	70.7	8301	PICALM	3	0	137581.0068	0	4
Q92574-1	Hamartin [OS=Homo sapiens]	129.7	7248	TSC1	2	0	244297.3737	3	4
Q5XX13-2	Isoform 2 of F-box/WD repeat-containing protein 10 [OS=Homo sapiens]	120.6	10517	FBXW10	1	0	223672.5828	0	4
P20340-1	Ras-related protein Rab-6A [OS=Homo sapiens]	23.6	5870	RAB6A	1	0	42846.98119	0	4
O95197	Reticulon-3 [OS=Homo sapiens]	112.5	10313	RTN3	3	0	211699.0088	0	4
P38606	V-type proton ATPase catalytic subunit A [OS=Homo sapiens]	68.3	523	ATP6V1A	2	0	126784.22	0	4
O95470	sphingosine-1-phosphate lyase 1 [OS=Homo sapiens]	63.5	8879	SGPL1	1	0	113792.5854	0	4
Q9UPQ9	Trinucleotide repeat-containing gene 6B protein [OS=Homo sapiens]	193.9	23112	TNRC6B	3	0	366558.3751	4	4
Q92783	signal transducing adapter molecule 1 [OS=Homo sapiens]	59.1	8027	STAM	1	0	107172.4949	0	4
O60749	Sorting nexin-2 [OS=Homo sapiens]	58.4	6643	SNX2	2	0	101334.7057	0	4
Q04721	Neurogenic locus notch homolog protein 2 [OS=Homo sapiens]	265.2	4853	NOTCH2	4	0	482033.9966	0	4
O95861-2	Isoform 2 of 3'(2'),5'-bisphosphate nucleotidase 1 [OS=Homo sapiens]	35.7	10380	BPNT1	1	0	62294.83319	0	4
P22102-1	trifunctional purine biosynthetic protein adenosine-3 [OS=Homo sapiens]	107.7	2618	GART	1	0	138198.5707	0	4
Q9Y6M7-7	Isoform 7 of Sodium bicarbonate cotransporter 3 [OS=Homo sapiens]	140.7	9497	SLC4A7	2	0	232889.3851	0	4
Q8N6H7	ADP-ribosylation factor GTPase-activating protein 2 [OS=Homo sapiens]	56.7	84364	ARFGAP2	2	0	95356.50526	1	4
O96005-1	Cleft lip and palate transmembrane protein 1 [OS=Homo sapiens]	76	1209	CLPTM1	1	0	119482.4262	0	4
P27694	Replication protein A 70 kDa DNA-binding subunit [OS=Homo sapiens]	68.1	6117	RPA1	1	0	25255.93164	0	4
Q9NP72-2	Isoform 2 of Ras-related protein Rab-18 [OS=Homo sapiens]	26.4	22931	RAB18	1	0	40562.42228	0	4
O00505	Importin subunit alpha-4 [OS=Homo sapiens]	57.8	3839	KPNA3	2	0	89074.22006	0	4
Q5JTV8	Torsin-1A-interacting protein 1 [OS=Homo sapiens]	66.2	26092	TOR1AIP1	24	0	98352.31488	0	4
P51159-1	Ras-related protein Rab-27A [OS=Homo sapiens]	24.9	5873	RAB27A	1	0	36697.3474	0	4
P46459	Vesicle-fusing ATPase [OS=Homo sapiens]	82.5	4905	NSF	2	0	122474.1436	0	4
Q9Y3E5	Peptidyl-tRNA hydrolase 2, mitochondrial [OS=Homo sapiens]	19.2	51651	PTRH2	1	0	29249.35028	0	4
Q86Y07	serine/threonine-protein kinase VRK2 [OS=Homo sapiens]	58.1	7444	VRK2	3	0	82235.55793	1	4
Q5F1R6-2	Isoform 2 of DnaJ homolog subfamily C member 21 [OS=Homo sapiens]	67.1	134218	DNAJC21	1	0	91983.87129	0	4
Q5T8D3-3	Isoform 3 of Acyl-CoA-binding domain-containing protein 5 [OS=Homo sapiens]	58.9	91452	ACBD5	14	0	42099.67423	2	4
P26374	Rab proteins geranylgeranyltransferase component A 2 [OS=Homo sapiens]	74	1122	CHML	1	0	102535.0482	0	4
Q96NT0	Coiled-coil domain-containing protein 115 [OS=Homo sapiens]	19.7	84317	CCDC115	2	0	27389.67953	0	4
P29144	Tripeptidyl-peptidase 2 [OS=Homo sapiens]	138.3	7174	TPP2	1	0	186831.3452	0	4
Q14678-1	KN motif and ankyrin repeat domain-containing protein 1 [OS=Homo sapiens]	147.2	23189	KANK1	3	0	201310.3578	0	4
P46937-9	Isoform 9 of Transcriptional coactivator YAP1 [OS=Homo sapiens]	54.9	10413	YAP1	1	0	55237.45227	2	4
Q08554-1	Desmocollin-1 [OS=Homo sapiens]	99.9	1823	DSC1	1	0	65082.14324	4	4
Q6P3W7	SCY1-like protein 2 [OS=Homo sapiens]	103.6	55681	SCYL2	2	0	133801.9522	1	4
P50502	Hsc70-interacting protein [OS=Homo sapiens]	41.3	6767	ST13	1	0	52564.08501	1	4
Q9H987	Synaptopodin 2-like protein [OS=Homo sapiens]	102.4	79933	SYNPO2L	1	0	140017.3586	0	4
Q8WZA0-2	Isoform 2 of Protein LZIC [OS=Homo sapiens]	23.9	84328	LZIC	5	0	30153.95571	0	4
P23759-1	Isoform 1 of Paired box protein Pax-7 [OS=Homo sapiens]	56.9	5081	PAX7	1	0	73375.16888	0	4
Q04695	Keratin, type I cytoskeletal 17 [OS=Homo sapiens]	48.1	3872	KRT17	5	0	29898.23814	0	4
Q08379	Golgin subfamily A member 2 [OS=Homo sapiens]	113	2801	GOLGA2	2	0	136666.9071	0	4
Q53EU6	Glycerol-3-phosphate acyltransferase 3 [OS=Homo sapiens]	48.7	84803	AGPAT9; GPAT3	1	0	58700.61091	1	4
Q9H4A5	Golgi phosphoprotein 3-like [OS=Homo sapiens]	32.7	55204	GOLPH3L	4	0	38615.08543	0	4
P07900-2	Isoform 2 of Heat shock protein HSP 90-alpha [OS=Homo sapiens]	98.1	3320	HSP90AA1	9	0	114821.0268	0	2
Q5SW79-1	Centrosomal protein of 170 kDa [OS=Homo sapiens]	175.2	9859	CEP170	2	0	208593.3292	0	4
P26196	Probable ATP-dependent RNA helicase DDX6 [OS=Homo sapiens]	54.4	1656	DDX6	2	0	61874.05469	0	4
Q9UH99-2	Isoform 2 of SUN domain-containing protein 2 [OS=Homo sapiens]	82.5	25777	SUN2	1	0	92000.39569	0	4
Q7Z3D4	LysM and putative peptidoglycan-binding domain-containing protein 3 [OS=Homo sapiens]	34.5	116068	LYSMD3	1	0	37770.90867	0	2
Q96AE4-2	Isoform 2 of Far upstream element-binding protein 1 [OS=Homo sapiens]	68.6	8880	FUBP1	1	0	79789.38826	1	4
Q96PY6-3	Isoform 3 of Serine/threonine-protein kinase Nek1 [OS=Homo sapiens]	145.7	4750	NEK1	1	0	154481.0345	0	4
Q8IYS0-1	GRAM domain-containing protein 1C [OS=Homo sapiens]	76	54762	GRAMD1C	2	0	78489.32125	0	4
P30086	phosphatidylethanolamine-binding protein 1 [OS=Homo sapiens]	21	5037	PEBP1	1	0	22260.70775	0	4
Q96BH1	E3 ubiquitin-protein ligase RNF25 [OS=Homo sapiens]	51.2	64320	RNF25	1	0	13616.31409	4	4
O95684	FGFR1 oncogene partner [OS=Homo sapiens]	43	11116	FGFR1OP	1	0	45720.70339	0	4
O00767	acyl-CoA desaturase [OS=Homo sapiens]	41.5	6319	SCD	1	0	40863.18845	0	4
O94966-5	Isoform 5 of Ubiquitin carboxyl-terminal hydrolase 19 [OS=Homo sapiens]	155.9	10869	USP19	4	0	159743.1702	0	4
Q8NEZ2	Vacuolar protein sorting-associated protein 37A [OS=Homo sapiens]	44.3	137492	VPS37A	2	0	44569.51551	0	4
Q02790	Peptidyl-prolyl cis-trans isomerase FKBP4 [OS=Homo sapiens]	51.8	2288	FKBP4	1	0	51219.12505	0	4
Q8TES7-6	Isoform 6 of Fas-binding factor 1 [OS=Homo sapiens]	127	85302	FBF1	1	0	127381.7656	1	4
Q9NUQ3-1	Gamma-taxilin [OS=Homo sapiens]	60.5	55787	TXLNG	1	0	58200.72219	0	4
Q9UNH7-1	Sorting nexin-6 [OS=Homo sapiens]	46.6	58533	SNX6	3	0	44099.87772	0	4
Q68DC2-4	Isoform 3 of Ankyrin repeat and SAM domain-containing protein 6 [OS=Homo sapiens]	92.3	203286	ANKS6	1	0	94745.80102	0	4
Q16625-1	Occludin [OS=Homo sapiens]	59.1	100506658	OCLN	2	0	54732.86026	0	4
Q86TI4-1	Isoform 1 of WD repeat-containing protein 86 [OS=Homo sapiens]	53	349136	WDR86	1	0	26303.18555	0	3
Q7Z2W4-3	Isoform 3 of Zinc finger CCCH-type antiviral protein 1 [OS=Homo sapiens]	67.5	56829	ZC3HAV1	23	0	32848.46415	0	4
P78527	DNA-dependent protein kinase catalytic subunit [OS=Homo sapiens]	468.8	5591	PRKDC	1	0	428770.9892	0	4
Q5SQN1-1	Synaptosomal-associated protein 47 [OS=Homo sapiens]	52.5	116841	SNAP47	2	0	48226.7616	0	4
P60842	Eukaryotic initiation factor 4A-I [OS=Homo sapiens]	46.1	1973	EIF4A1	1	0	41471.6112	0	4
Q96QD5-1	DEP domain-containing protein 7 [OS=Homo sapiens]	58.3	91614	DEPDC7	1	0	52409.46528	0	4
Q96EY5-1	Multivesicular body subunit 12A [OS=Homo sapiens]	28.8	93343	FAM125A; MVB12A	1	0	27713.39294	1	4
Q96M27-2	Isoform 2 of Protein PRRC1 [OS=Homo sapiens]	48.5	133619	PRRC1	1	0	47009.60805	2	4
Q4VCS5	Angiomotin [OS=Homo sapiens]	118	154796	AMOT	2	0	108455.6167	0	4
Q8WXF7	Atlastin-1 [OS=Homo sapiens]	63.5	51062	ATL1	2	0	54763.49152	0	4
O75475-1	PC4 and SFRS1-interacting protein [OS=Homo sapiens]	60.1	11168	PSIP1	1	0	51081.2973	0	4
Q9UKA4	A-kinase anchor protein 11 [OS=Homo sapiens]	210.4	11215	AKAP11	3	0	182160.2375	0	4
Q8WUM0	Nuclear pore complex protein Nup133 [OS=Homo sapiens]	128.9	55746	NUP133	5	0	109494.5876	0	4
P62495	Eukaryotic peptide chain release factor subunit 1 [OS=Homo sapiens]	49	2107	ETF1	2	0	40282.35719	0	4
P38435-1	vitamin K-dependent gamma-carboxylase [OS=Homo sapiens]	87.5	2677	GGCX	1	0	69636.98006	0	4
Q8IZH2-1	5'-3' exoribonuclease 1 [OS=Homo sapiens]	194	54464	XRN1	4	0	153937.5628	0	4
Q5JWF2-1	Guanine nucleotide-binding protein G(S) subunit alpha isoforms XLas [OS=Homo sapiens]	111	2778	GNAS	1	0	93343.22858	1	4
Q86V85	Integral membrane protein GPR180 [OS=Homo sapiens]	49.4	160897	GPR180	1	0	39330.30666	0	4
P35869	Aryl hydrocarbon receptor [OS=Homo sapiens]	96.1	196	AHR	1	0	73192.85932	0	4
Q96D09	G-protein coupled receptor-associated sorting protein 2 [OS=Homo sapiens]	93.7	114928; 100528062	GPRASP2; ARMCX5-GPRASP2	1	0	54758.08621	0	4
Q9NT62	ubiquitin-like-conjugating enzyme ATG3 [OS=Homo sapiens]	35.8	64422	ATG3	1	0	26763.80048	0	4
Q12846	Syntaxin-4 [OS=Homo sapiens]	34.2	6810	STX4	1	0	24679.83485	0	4

Q9NRY4	Rho GTPase-activating protein 35 [OS=Homo sapiens]	170.4	2909	ARHGAP35	4	0	123767.7389	0	4
Q15084-2	Isoform 2 of Protein disulfide-isomerase A6 [OS=Homo sapiens]	53.9	10130	PDIA6	1	0	40087.65088	0	3
P59046-7	Isoform 7 of NACHT, LRR and PYD domains-containing protein 12 [OS=Homo sapiens]	120.3	91662	NLRP12	1	0	84421.0558	0	4
Q96JA3	pleckstrin homology domain-containing family A member 8 [OS=Homo sapiens]	58.2	84725	PLEKHA8	1	0	41191.40955	0	4
Q9BRX8-1	Redox-regulatory protein FAM213A [OS=Homo sapiens]	25.7	84293	FAM213A	2	0	17613.91794	0	4
Q53H82	Endoribonuclease LACTB2 [OS=Homo sapiens]	32.8	51110	LACTB2	1	0	15926.08603	0	4
P31949	protein S100-A11 [OS=Homo sapiens]	11.7	6282	S100A11	1	0	5719.303973	0	4
Q6IBS0	Twinfilin-2 [OS=Homo sapiens]	39.5	11344	TWF2	3	0	24671.76009	0	4
Q9Y450-1	HBS1-like protein [OS=Homo sapiens]	75.4	10767	HBS1L	1	0	47129.06376	0	4
O15014	zinc finger protein 609 [OS=Homo sapiens]	151.1	23060	ZNF609	1	0	96091.29031	0	4
Q8N2H4	protein SYS1 homolog [OS=Homo sapiens]	17.6	90196	SYS1	1	0	10026.52192	1	4
O75534-4	Isoform 4 of Cold shock domain-containing protein E1 [OS=Homo sapiens]	93.7	7812	CSDE1	1	0	53993.80314	3	4
Q9NWZ3-1	Interleukin-1 receptor-associated kinase 4 [OS=Homo sapiens]	51.5	51135	IRAK4	4	0	28356.99537	0	4
P15260	Interferon gamma receptor 1 [OS=Homo sapiens]	54.4	3459	IFNGR1	2	0	30343.64156	0	4
P05388	60S acidic ribosomal protein P0 [OS=Homo sapiens]	34.3	6175	RPLP0	1	0	19354.08024	0	4
Q13620	Cullin-4B [OS=Homo sapiens]	103.9	8450	CUL4B	1	0	28138.73901	0	4
Q8NFP9-1	Neurobeachin [OS=Homo sapiens]	327.6	26960	NBEA	3	0	176038.1797	1	4
Q6GQQ9-1	OTU domain-containing protein 7B [OS=Homo sapiens]	92.5	56957	OTUD7B	1	0	48689.29984	0	4
P53990-5	Isoform 5 of IST1 homolog [OS=Homo sapiens]	41.5	9798	IST1	1	0	21373.35656	0	4
Q6PJW8	Consortin [OS=Homo sapiens]	79.5	163882	CNST	4	0	40238.94211	0	4
Q6NUM9-1	all-trans-retinol 13,14-reductase [OS=Homo sapiens]	66.8	54884	RETSAT	2	0	33806.82559	0	4
Q8TDG2	actin-related protein T1 [OS=Homo sapiens]	41.7	139741	ACTRT1	1	0	20248.25897	0	4
P08779	Keratin, type I cytoskeletal 16 [OS=Homo sapiens]	51.2	3868	KRT16	8	0	18781.38213	1	4
Q9H0E7	Ubiquitin carboxyl-terminal hydrolase 44 [OS=Homo sapiens]	81.1	84101	USP44	1	0	37225.29427	0	4
Q8N434-1	Putative transporter SVOPL [OS=Homo sapiens]	54	136306	SVOPL	1	0	24875.05384	0	4
Q07617	Sperm-associated antigen 1 [OS=Homo sapiens]	103.6	6674	SPAG1	3	0	45489.1338	0	4
O43379-4	Isoform 4 of WD repeat-containing protein 62 [OS=Homo sapiens]	166.4	284403	WDR62	1	0	73140.70585	1	4
Q8N3C0	Activating signal cointegrator 1 complex subunit 3 [OS=Homo sapiens]	251.3	10973	ASCC3	3	0	103370.2957	0	4
Q9UNH6-3	Isoform 3 of Sorting nexin-7 [OS=Homo sapiens]	51.6	51375	SNX7	2	0	20995.31111	0	4
O95433	activator of 90 kDa heat shock protein ATPase homolog 1 [OS=Homo sapiens]	38.3	10598	AHSA1	1	0	14705.42237	0	4
O75663	TIP41-like protein [OS=Homo sapiens]	31.4	261726	TIPRL	2	0	11504.24239	0	4
Q8WXH0-2	Isoform 2 of Nesprin-2 [OS=Homo sapiens]	798.4	23224	SYNE2	10	0	271252.7071	0	4
O60488-1	Long-chain-fatty-acid--CoA ligase 4 [OS=Homo sapiens]	79.1	2182	ACSL4	3	0	27456.89981	0	4
O75312	Zinc finger protein ZPR1 [OS=Homo sapiens]	50.9	8882	ZNF259; ZPR1	1	0	12973.41157	4	4
P53618	Coatomer subunit beta [OS=Homo sapiens]	107.1	1315	COPB1	1	0	34515.06286	0	4
P04083	annexin A1 [OS=Homo sapiens]	38.7	301	ANXA1	1	0	11277.34006	0	4
Q8NF37	Lysophosphatidylcholine acyltransferase 1 [OS=Homo sapiens]	59.1	79888	LPCAT1	1	0	4181.97868	0	4
P35555	Fibrillin-1 [OS=Homo sapiens]	312	2200	FBN1	1	0	89665.72088	0	4
Q8TBZ3-7	Isoform 7 of WD repeat-containing protein 20 [OS=Homo sapiens]	66.5	91833	WDR20	1	0	17919.3922	0	4
P27105	erythrocyte band 7 integral membrane protein [OS=Homo sapiens]	31.7	2040	STOM	1	0	7778.076433	0	4
Q969S3	Zinc finger protein 622 [OS=Homo sapiens]	54.2	90441	ZNF622	1	0	12652.36661	1	4
Q96RN5	Mediator of RNA polymerase II transcription subunit 15 [OS=Homo sapiens]	86.7	51586	MED15	1	0	14345.69033	0	4
Q3YEC7-2	Isoform 2 of Rab-like protein 6 [OS=Homo sapiens]	79.6	55684	RABL6	1	0	17037.68056	0	4
Q13148-1	TAR DNA-binding protein 43 [OS=Homo sapiens]	44.7	23435	TARDBP	2	0	2398.61975	3	4
P53992	Protein transport protein sec24c [OS=Homo sapiens]	118.2	9632	SEC24C	1	0	24157.68935	1	4
Q96EB1-2	Isoform 2 of Elongator complex protein 4 [OS=Homo sapiens]	58.7	26610	ELP4	1	0	11421.97047	0	4
Q14410	glycerol kinase 2 [OS=Homo sapiens]	60.6	2712	GK2	1	0	11477.51793	2	4
Q68DQ2	very large A-kinase anchor protein [OS=Homo sapiens]	330.4	131544	CRYBG3	5	0	60092.77025	0	4
Q8WU76-1	Sec1 family domain-containing protein 2 [OS=Homo sapiens]	75.1	152579	SCFD2	1	0	13654.03676	0	4
Q96CP6-1	GRAM domain-containing protein 1A [OS=Homo sapiens]	80.6	57655	GRAMD1A	1	0	14329.72032	0	4
Q92997	segment polarity protein dishevelled homolog DVL-3 [OS=Homo sapiens]	78	1857	DVL3	1	0	14008.53306	0	2
Q9Y5X1	Sorting nexin-9 [OS=Homo sapiens]	66.6	51429	SNX9	1	0	11288.62188	0	4
Q96G28-2	Isoform 2 of Cilia- and flagella-associated protein 36 [OS=Homo sapiens]	41.9	112942	CCDC104; CFAP36	1	0	6662.904052	0	4
Q13561-2	Isoform 2 of Dynactin subunit 2 [OS=Homo sapiens]	44.8	10540	DCTN2	1	0	1662.147095	0	4
Q93009	Ubiquitin carboxyl-terminal hydrolase 7 [OS=Homo sapiens]	128.2	7874	USP7	2	0	18486.86606	0	4
Q9NW68-3	Isoform 3 of BSD domain-containing protein 1 [OS=Homo sapiens]	51.1	55108	BSDC1	1	0	7626.953075	0	4
Q92667	A-kinase anchor protein 1, mitochondrial [OS=Homo sapiens]	97.3	8165	AKAP1	2	0	6630.046069	0	4
P18206	Vinculin [OS=Homo sapiens]	123.7	7414	VCL	2	0	17098.44475	0	4
Q9NZJ5	Eukaryotic translation initiation factor 2-alpha kinase 3 [OS=Homo sapiens]	125.1	9451	EIF2AK3	1	0	16051.40347	0	4
O00186	syntaxin-binding protein 3 [OS=Homo sapiens]	67.7	6814	STXBP3	1	0	3956.301192	0	4
Q9P2E3-1	NFX1-type zinc finger-containing protein 1 [OS=Homo sapiens]	220.1	57169	ZNFX1	1	0	25756.15895	0	4
Q8IWQ3-1	Serine/threonine-protein kinase BRSK2 [OS=Homo sapiens]	81.6	9024	BRSK2	1	0	5106.33596	0	4
Q6NUQ1	RAD50-interacting protein 1 [OS=Homo sapiens]	90.6	60561	RINT1	1	0	10086.6595	0	4
Q96HR8	H/ACA ribonucleoprotein complex non-core subunit NAF1 [OS=Homo sapiens]	53.7	92345	NAF1	1	0	6150.583742	2	4
P11717	Cation-independent mannose-6-phosphate receptor [OS=Homo sapiens]	274.2	3482	IGF2R	1	0	30542.40317	0	2
Q9BPX3	Condensin complex subunit 3 [OS=Homo sapiens]	114.3	64151	NCAPG	1	0	5982.211934	1	4
P13010	X-ray repair cross-complementing protein 5 [OS=Homo sapiens]	82.7	7520	XRCC5	1	0	3921.700719	0	4
O94874-1	E3 UFM1-protein ligase 1 [OS=Homo sapiens]	89.5	23376	UFL1	1	0	2016.116211	0	4
P46531	Neurogenic locus notch homolog protein 1 [OS=Homo sapiens]	272.3	4851	NOTCH1	1	0	24218.72914	0	4
Q9BRR8	G patch domain-containing protein 1 [OS=Homo sapiens]	103.3	55094	GPATCH1	1	0	1889.898193	0	4
O14974	Protein phosphatase 1 regulatory subunit 12A [OS=Homo sapiens]	115.2	4659	PPP1R12A	2	0	6652.048071	0	4
Q9UL51	Potassium/sodium hyperpolarization-activated cyclic nucleotide-gated channel 2 [OS=Homo sapiens]	96.9	610	HCN2	1	0	2284.95798	0	4
P15924-1	Desmoplakin [OS=Homo sapiens]	331.6	1832	DSP	1	0	5881.237747	0	4
Q5RHP9	glutamate-rich protein 3 [OS=Homo sapiens]	168.4	127254	C1orf173; ERICH3	1	0	2508.935327	0	4
A2RRP1-1	neuroblastoma-amplified sequence [OS=Homo sapiens]	268.4	51594	NBAS	1	0	4861.701591	0	4
Q9UQP3	tenascin-N [OS=Homo sapiens]	143.9	63923	TNN	1	0	0	0	4
Q8IXJ6-2	Isoform 2 of NAD-dependent protein deacetylase sirtuin-2 [OS=Homo sapiens]	39.5	22933	SIRT2	1	0	0	0	4
Q9H0C8	Integrin-linked kinase-associated serine/threonine phosphatase 2C [OS=Homo sapiens]	42.9	80895	ILKAP	1	0	0	0	4
P04406-1	glyceraldehyde-3-phosphate dehydrogenase [OS=Homo sapiens]	36	2597	GAPDH	1	0	0	0	4
P40227-1	T-complex protein 1 subunit zeta [OS=Homo sapiens]	58	908	CCT6A	1	0	0	0	4
Q9H981-1	Actin-related protein 8 [OS=Homo sapiens]	70.4	93973	ACTR8	1	0	0	0	4
Q96CM8-2	Isoform 2 of Acyl-CoA synthetase family member 2, mitochondrial [OS=Homo sapiens]	70.6	80221	ACSF2	1	0	0	0	4
P09543-1	2',3'-cyclic-nucleotide 3'-phosphodiesterase [OS=Homo sapiens]	47.5	1267	CNP	1	0	0	0	3
P10398-1	serine/threonine-protein kinase A-Raf [OS=Homo sapiens]	67.5	369	ARAF	1	0	0	0	3
Q92547	DNA topoisomerase 2-binding protein 1 [OS=Homo sapiens]	170.6	11073	TOPBP1	1	0	0	0	2
P56962	Syntaxin-17 [OS=Homo sapiens]	33.4	55014	STX17	1	0	0	0	2
Q96T51	RUN and FYVE domain-containing protein 1 [OS=Homo sapiens]	79.8	80230	RUFY1	1	0	0	0	2
Q3KQU3	MAP7 domain-containing protein 1 [OS=Homo sapiens]	92.8	55700	MAP7D1	1	0	0	0	2
Q9H6S0	Probable ATP-dependent RNA helicase YTHDC2 [OS=Homo sapiens]	160.1	64848	YTHDC2	1	0	0	0	1
P98170	E3 ubiquitin-protein ligase XIAP [OS=Homo sapiens]	56.6	331	XIAP	1	0	0	0	1
Q9P0L0-2	Isoform 2 of Vesicle-associated membrane protein-associated protein A [OS=Homo sapiens]	32.6	9218	VAPA	11	0	0	0	1
P61081	NEDD8-conjugating enzyme Ubc12 [OS=Homo sapiens]	20.9	9040	UBE2M	1	0	0	0	1
Q12792-3	Isoform 3 of Twinfilin-1 [OS=Homo sapiens]	41	5756	TWF1	1	0	0	0	1
Q969M1	Mitochondrial import receptor subunit TOM40B [OS=Homo sapiens]	33.9	84134	TOMM40L	1	0	0	0	1
P29401-2	Isoform 2 of Transketolase [OS=Homo sapiens]	68.8	7086	TKT	1	0	0	0	1
Q9UI15	Transgelin-3 [OS=Homo sapiens]	22.5	29114	TAGLN3	2	0	0	0	1
Q15750-1	TGF-beta-activated kinase 1 and MAP3K7-binding protein 1 [OS=Homo sapiens]	54.6	10454	TAB1	1	0	0	0	1
Q9H2B2	Synaptotagmin-4 [OS=Homo sapiens]	47.9	6860	SYT4	1	0	0	0	1
Q9Y230	RuvB-like 2 [OS=Homo sapiens]	51.1	10856	RUVBL2	1	0	0	0	1
P32969	60S ribosomal protein L9 [OS=Homo sapiens]	21.9	6133	RPL9	1	0	0	0	1
Q5T8P6	RNA-binding protein 26 [OS=Homo sapiens]	113.5	64062	RBM26	1	0	0	0	1
O60256-1	Phosphoribosyl pyrophosphate synthase-associated protein 2 [OS=Homo sapiens]	40.9	5636	PRPSAP2	1	0	0	0	1
P07737	profilin-1 [OS=Homo sapiens]	15	5216	PFN1	1	0	0	0	1
O75381-1	Peroxisomal membrane protein PEX14 [OS=Homo sapiens]	41.2	5195	PEX14	1	0	0	0	1
Q12769	nuclear pore complex protein Nup160 [OS=Homo sapiens]	162	23279	NUP160	1	0	0	0	1
Q15390	Mitochondrial fission regulator 1 [OS=Homo sapiens]	37	9650	MTFR1	2	0	0	0	1
O75970	multiple PDZ domain protein [OS=Homo sapiens]	221.5	8777	MPDZ	1	0	0	0	1
Q14149	MORC family CW-type zinc finger protein 3 [OS=Homo sapiens]	107	23515	MORC3	1	0	0	0	1
O60244	Mediator of RNA polymerase II transcription subunit 14 [OS=Homo sapiens]	160.5	9282	MED14	1	0	0	0	1
Q9UNF1	Melanoma-associated antigen D2 [OS=Homo sapiens]	64.9	10916	MAGED2	1	0	0	0	1
Q38SD2	Leucine-rich repeat serine/threonine-protein kinase 1 [OS=Homo sapiens]	225.2	79705	LRRK1	1	0	0	0	1
O75473	Leucine-rich repeat-containing G-protein coupled receptor 5 [OS=Homo sapiens]	99.9	8549	LGR5	1	0	0	0	1
Q86UP2-2	Isoform 2 of Kinectin [OS=Homo sapiens]	149.5	3895	KTN1	95	0	0	0	1
Q86VZ6	juxtaposed with another zinc finger protein 1 [OS=Homo sapiens]	27.1	221895	JAZF1	1	0	0	0	1
P23458	Tyrosine-protein kinase JAK1 [OS=Homo sapiens]	133.2	3716	JAK1	1	0	0	0	1
Q14571-1	Inositol 1,4,5-trisphosphate receptor type 2 [OS=Homo sapiens]	307.9	3709	ITPR2	1	0	0	0	1
O43736-1	Integral membrane protein 2A [OS=Homo sapiens]	29.7	9452	ITM2A	1	0	0	0	1
Q9NQS7	Inner centromere protein [OS=Homo sapiens]	105.4	3619	INCENP	1	0	0	0	1
A0A075B6S5	immunoglobulin kappa variable 1-27 [OS=Homo sapiens]	12.7	28935	IGKV1-27	1	0	0	0	1
Q53GQ0	Very-long-chain 3-oxoacyl-CoA reductase [OS=Homo sapiens]	34.3	51144	HSD17B12	1	0	0	0	1
Q9Y3E1	Hepatoma-derived growth factor-related protein 3 [OS=Homo sapiens]	22.6	50810	HDGFRP3; HDGFL3	1	0	0	0	1
P02100	Hemoglobin subunit epsilon [OS=Homo sapiens]	16.2	3046	HBE1	1	0	0	0	1
P63244	Receptor of activated protein C kinase 1 [OS=Homo sapiens]	35.1	10399	GNB2L1; RACK1	1	0	0	0	1
Q6Y7W6-3	Isoform 2 of GRB10-interacting GYF protein 2 [OS=Homo sapiens]	152.3	26058	GIGYF2	1	0	0	0	1
P57678	Gem-associated protein 4 [OS=Homo sapiens]	120	50628	GEMIN4	1	0	0	0	1
P20042	Eukaryotic translation initiation factor 2 subunit 2 [OS=Homo sapiens]	38.4	8894	EIF2S2	1	0	0	0	1
Q9NSV4-3	Protein diaphanous homolog 3 [OS=Homo sapiens]	136.8	81624	DIAPH3	1	0	0	0	1
Q2VPK5-1	Cytoplasmic tRNA 2-thiolation protein 2 [OS=Homo sapiens]	56.1	348180	CTU2	1	0	0	0	1
P17927	Complement receptor type 1 [OS=Homo sapiens]	223.5	1378	CR1	1	0	0	0	1
P12111	Collagen alpha-3(VI) chain [OS=Homo sapiens]	343.5	1293	COL6A3	1	0	0	0	1
Q8NFW1	Collagen alpha-1(XXII) chain [OS=Homo sapiens]	161	169044	COL22A1	1	0	0	0	1
P02452	Collagen alpha-1(I) chain [OS=Homo sapiens]	138.9	1277	COL1A1	1	0	0	0	1
Q7Z3S7-5	Isoform 5 of Voltage-dependent calcium channel subunit alpha-2/delta-4 [OS=Homo sapiens]	129.9	93589	CACNA2D4	1	0	0	0	1
P00736	Complement C1r subcomponent [OS=Homo sapiens]	80.1	715	C1R	1	0	0	0	1
Q7Z6K5-2	Isoform C15orf38-AP3S2 of Arpin [OS=Homo sapiens]	43.9	100526783; 348110; 10239	C15orf38-AP3S2; C15orf38; ARPIN; AP3S2	1	0	0	0	1
O75185-3	Isoform 3 of Calcium-transporting ATPase type 2C member 2 [OS=Homo sapiens]	106.2	9914	ATP2C2	1	0	0	0	1
O75915	PRA1 family protein 3 [OS=Homo sapiens]	21.6	10550	ARL6IP5	1	0	0	0	1
Q9NW15-5	Isoform 5 of Anoctamin-10 [OS=Homo sapiens]	72.1	55129	ANO10	2	0	0	0	1
P49419	Alpha-aminoadipic semialdehyde dehydrogenase [OS=Homo sapiens]	58.5	501	ALDH7A1	1	0	0	0	1
Q12904-2	Isoform 2 of Aminoacyl tRNA synthase complex-interacting multifunctional protein 1 [OS=Homo sapiens]	37	9255	AIMP1	1	0	0	0	1
Q9ULC5-3	Isoform 2 of Long-chain-fatty-acid--CoA ligase 5 [OS=Homo sapiens]	82.2	51703	ACSL5	1	0	0	0	1
P53396-1	ATP-citrate synthase [OS=Homo sapiens]	120.8	47	ACLY	1	0	0	0	1
Q6ZU15	Septin-14 [OS=Homo sapiens]	50	346288	SEPT14	1	0	0	0	1
