# Supplementary material for: SNARE protein USE1 is involved in the glycosylation and the expression of mumps virus fusion protein and important for viral propagation
Source: PLoS Pathog. 2022 Dec 8;18(12):e1010949. doi: 10.1371/journal.ppat.1010949 (PMC9731409; doi:10.1371/journal.ppat.1010949)
Supplement: Attachment — Submitted filename: Liu&KatohPLoSPathog_Answers_R1.docx [file ppat.1010949.s007.docx]

**Responses to the Editor:**

**Specifically, Reviewer 3 raised valid concerns about over-interpretation and incomplete/incorrect interpretation regarding the cell biological function of USE1 (e.g., whether the effects on glycosylation are indirect as a result of its function (or lack thereof in KD cells) in Golgi to ER retrograde transport as opposed to direct effects on glycosyation per se). Please pay attention to these various interpretation issues.**

Thank you very much for the comments. We have carefully addressed all the points raised by reviewer #3.

**In addition, Reviewer 1 raised valid concerns regarding current standards (& best practices) for publication of studies using Mass Spec ID.  While it might not be possible to redo the experiments in triplicates using the controls that have been suggested, please provide the additional information requested and any additional analyses that will help readers to understand the power AND limitations of the methods used. Note that standard practice requires deposition of raw Mass Spec data in repositories such as PRIDE Archive, MassIVE, jPOST and iProX (see ProteomeXchange consortium).**

We have added detailed descriptions of proximity labeling proteomics to the relevant parts of the manuscript. Although not all of the proposed experiments were performed, we have answered reviewer #1’s points as much as possible. We have also deposited the raw MS data in PRIDE (Project accession ID: PXD035151).

Other such claims in the manuscript were revised in response to reviewer comments.

Because the affiliations of two authors changed after our initial submission, we have revised the title page accordingly.

**Responses to Reviewer #1:**

**The major weaknesses of the manuscript relate to the description and application of proximity labeling proteomics that led to identification of USE1.**

We have revised the manuscript as described below to address the specific points highlighted by Reviewer #1.

**The authors provide no negative control for the proximity labeling experiment. Proximity labeling technology is known to yield very high background, and that seems to be the case here considering the authors identified 638 protein in this analysis that almost certainly do not all interact with F/HN. At the very least, a control experiment where cells not supplemented with biotin were subjected to the same strep pulldown and MS analysis would allow subtraction of proteins non-specifically bindings to strep beads. Even better would be to provide a control experiment with half of turboID fused to a protein that does not interact with F/HN (GFP, for example).**

Thank you very much for the suggestions about appropriate negative controls. As noted in the reviewer’s comments, proximity labeling technology produces data with a high background signal, so it is therefore necessary to set up appropriate negative controls for experiments performed with this method. In the present study, we used wildtype viral proteins without split-TurboID as a negative control to eliminate nonspecific bindings to strep beads. We have added information about our negative control in the main text (lines 441–443 and 806–813) and in S2 Fig of the revised manuscript.

**These experiments should be performed with at least three biological replicates, but it appears that these experiments were not replicated at all. Including replicates and negative controls will enable the authors to perform a statistical protein interaction analysis such as SAINT to calculate an interaction score for each protein based on the peptide counts or intensity values in the test samples compared to negative controls. These scores are extremely useful to rank the data and quickly identify the top hits instead of providing an unranked list of 638 proteins.**
Thank you very much for the comments about biological replicates. As shown in the S2 Fig in the revised manuscript, we prepared duplicates for each sample (control and split-TurboID), and an MS analysis was performed twice per duplicate in this study. For the analysis of proteomic datasets, we calculated the average of the detection intensities (protein abundance) obtained from each sample (control and split-TurboID), extracted factors that were detected only in the split-TurboID sample or factors with an intensity ratio (TurboID/control) of ≥10-fold, and identified 641 proteins as being significantly enriched in the split-TurboID-expressing cells. We have added descriptions of these points in lines 128–133 and 806–813 and in S2 Fig of the revised manuscript.

**The description of the proteomics analysis is not acceptable for publication. The methods section merely states that samples were “subjected to liquid chromatography-tandem mass spectrometry analysis”. This section should provide a description of the instrument used, a detailed description of the acquisition method, and a detailed description of the settings used for Proteome Discoverer for proteomics data analysis.**

In accordance with the reviewer’s suggestion, we have added more information about the mass spectrometry analysis in the revised manuscript, lines 464–475 and 479–482.

**Table S2 containing the proteomics results should provide metrics of protein identifications such as the number of peptides identified per protein and the label-free quantification intensity of the proteins.**

Thank you very much for this important comment. In accordance with the reviewer’s suggestion, we have added more information about the protein abundance (detection intensity), the number of peptides, and the number of samples in which peptides were detected, in S1 Table of the revised manuscript.

**Finally, it is standard practice for authors to deposit raw mass spectrometry data in a repository such as PRIDE.**

Thank you very much for this important comment. We have deposited the raw MS data in PRIDE (Project accession ID: PXD035151). We have also added a description of this in the revised manuscript, lines 552–554.

**Responses to Reviewer #2:**

**1. In the Introduction section, it would be helpful to provide the reader with some introduction to the split-TurboID approach that forms the basis for this study. I.e., how these proximity-based strategies work in general; advantages as compared to traditional affinity purifications; difference between BioID and Turbo-ID; etc.**

Thank you very much for this important comment. In accordance with the reviewer’s suggestion, we have added a new paragraph in the introduction section to explain proximity labeling, especially with regard to the points raised by the reviewer. We have modified the main text in lines 81–94 of the revised manuscript.

**2. Authors state that insertion of split-TurboID was tolerated, but reduced VLP production by about 10-fold. On the other hand, when the complete TurboID was added to N, M, or F, no VLPs could be produced. It would be helpful to show one of these negative VLP results as a control, to confirm that the 10-fold reduced VLP production observed in Fig. 1C is meaningful in comparison to this true negative, and thus represents more than background signal.**
In accordance with the reviewer’s suggestion, we have added more VLP assay data in S1 Fig of the revised manuscript. As shown in S1 Fig, when the complete TurboID was introduced into the C-terminal of M protein, no band corresponding to the M/TurboID protein was detected. We have modified the main text in lines 109-110 and 802–804.

**3. For Table S1, it is not clear how this list has been sorted or if the order has any meaning.**
Thank you very much for this important comment. To address the issue raised by the reviewer, we have added more information about the protein abundance (detection intensity), number of peptides, and number of samples in which peptides were detected into S1 Table of the revised manuscript. We then sorted the proteins by their abundance ratios (TurboID/control).

**4. Fig. 3A. The 13 that showed significant decreases in AcGFP expression should somehow be indicated directly on the figure.**
In accordance with the reviewer’s suggestion, we changed the colors to identify the 13 factors we selected in Fig 3A and modified the main text, lines 752–753. Although it appears that the factors with decreased AcGFP signals were not necessarily selected, the data displayed in Fig 3A is the result of only one experiment, and the 13 factors were selected after conducting the same experiment several times and examining the reproducibility.

**5. From authors’ brief description of Fig. 3B in the Results section, one could get the impression that the 13 candidates broke neatly into two categories - five that had significant effects on MuV replication, and eight that didn’t. In reality the data is more complex. In some cases there are striking differences between results obtained in A549 cells vs. Vero cells. Also, the magnitudes of viral replication defects vary significantly. This is tacitly acknowledged in that siLYPLA2 was not included in the five, as it results in only modest replication defects. But its exclusion makes authors’ statement starting on Line 158 untrue. This entire paragraph needs to be re-written to give readers a more complete description and understanding of the data, and to more accurately convey what the criteria were for selecting the five.**
Thank you very much for this important comment. In accordance with the reviewer’s suggestion, we have rewritten lines 170–178 of the revised manuscript to convey an accurate interpretation of the data. We selected five factors whose knockdown significantly reduced the level of viral propagation in both A549 and Vero cells without causing cytotoxicity.

**6. Fig. 4E is missing the legend that shows which colored line corresponds to which virus.**
Thank you very much. We have added a description of each colored line in lines 772–773 and 247 of the main text and in Fig. 5C of the revised manuscript.

**7. Fig. S3C shows clear differences in N protein accumulation after BET1 or USE1 knockdown. Quantification of these important results should be shown.**
Thank you very much for this important comment. In accordance with the reviewer’s suggestion, we have added data on the relative band intensity in Fig 5D and modified the main text in lines 205-207 and 774–776 of the revised manuscript.

**8. Throughout Fig. 5 when analyzing the F proteins with different mobilities on the blots, it should be made more clear whether what we are looking at is cleaved F or uncleaved F.**
Thank you very much for this important comment. We expressed the uncleaved F protein, whose multibasic cleavage motif (R-H-K-R-F-A) was mutated to N-H-N-R-F-A, in 293T cells. As shown in Fig 5D in the revised manuscript, a ~75-kDa band corresponding to the uncleaved F protein (F_0_) was detected. Therefore, the ~60-kDa band detected in the cells expressing the wildtype F protein was considered to be cleaved F protein (F_1_). We have modified lines 228–234, 382–383, and 788–791 in the main text of the revised manuscript.

**9. Please provide a brief description of the dual split protein assay system and how it works in the Results section for the benefit of readers who are unfamiliar with it.**
We have added the requested description in the main text, lines 218-224, of the revised manuscript.

**10. Results and/or Figure legend 5 should briefly state what approach has been used to measure cell surface transport of F.**

We have added the requested description in the main text, lines 256–260, of the revised manuscript.

**Responses to Reviewer #3:**

**1. The authors suggest that the detailed molecular mechanisms of N-linked glycosylation of paramyxovirus F proteins is poorly understood – but this seems like an overstatement. N-linked glycosylation is a highly conserved process for all proteins receiving these modifications, and a large set of the host enzymes involved in this process has been identified. Numerous studies have examined the effect of glycan mutants in paramyxovirus fusion proteins on changes in folding, trafficking, etc. This statement should be modified to better acknowledge the true state of the field.**

Thank you very much for this important comment. As the reviewer pointed out, our description “the detailed molecular mechanisms of N-linked glycosylation of paramyxovirus F proteins and the host factors in this process have been poorly elucidated” in the introduction section of the initially reviewed manuscript was an overstatement. In accordance with the reviewer’s suggestion, we have modified the text in lines 74–80 of the revised manuscript.

**2. There is a very large difference in the effect of siRNA knock-down between A549 and Vero cells for a number of the factors tested in Figure 3, including for USE1, that is the focus of the paper, though the authors do not really comment on this.**

Thank you very much for this important comment. In accordance with the reviewer’s and another reviewer’s comments, we have rewritten lines 170–178 of the revised manuscript to better reflect an accurate interpretation of the data.

**Why might the effect be so different between the two cells lines? And if USE1 is so critical for mumps infection – why is the effect so small in Vero cells?**

Thank you very much for the critical comment. In accordance with the reviewer’s below comment (No. 7), we have evaluated the glycosylation of the F protein in USE1-knockdown A549 and Vero cells. As shown in Fig 6H of the revised manuscript, the expression of F protein in A549 cells was significantly decreased by USE1-knockdown, whereas the expression level and the glycosylation of F protein in Vero cells were not affected by USE1-knockdown. The importance of each factor may differ between A549 cells and Vero cells, as exemplified by USE1. We have added a description and discussion in lines 264–269, 329–338, and 796–798 of the revised manuscript.

**3. In Figures 4B and 4C, the authors look a localization for F compared to BET1 and USE1. Why was the first examination done at 12 hours post infection? Given that F much utilize the secretory pathway for expression, modification and trafficking, it would be expected that it would be present in those regions much earlier in the infection process.**

Although we had investigated the localization of the F protein at 6 and 9 hpi, no specific signal corresponding to the F protein was detected by the anti-F antibody used in this study. Thus, we showed the data of 12, 18, and 24 hpi in Fig 4B and 4C.

**In addition, while there is certainly close localization of F with these proteins, in most cases the signal appears to be close together, rather than truly co-localized, as there appears to be very little yellow signal in the merge. Quantitative assessment of co-localization would be helpful in better assessing this.**

Thank you very much for this important comment. In accordance with the reviewer’s suggestion, we have analyzed the color profile of our IFA data (Fig 4B and 4C) by using ImageJ software. As shown in S5 Fig of the revised manuscript, it was confirmed that the F protein localized close to or partially co-localized with USE1 in the infected cells, whereas the co-localization of F protein and BET1 was not clear. We have modified the main text in lines 197–201 and 825–827 of the revised manuscript.

**Finally – the cellular location where the signals are close to each other is important but not commented on. BET1 and USE1 are ER/Golgi proteins, and that appears consistent with their localization in these images – suggesting that they have a role during F folding and trafficking in the early secretory pathway, but not a role later on in viral budding. The authors should more carefully examine their images and discuss these results in more depth.**

Thank you very much for the critical comment. In accordance with the reviewer’s suggestion, we have modified the main text in lines 197–201 and 301–309 of the revised manuscript.

**4. It would be very helpful to put the viral protein level figure (currently Figure S3A-C) into the main text, potentially in Figure 4, as this is likely the reason why viral titers eventually reduce.**

Thank you very much for your helpful advice. In accordance with the reviewer’s suggestion, we have moved data of S3 Fig in the initially reviewed manuscript to Fig 5B–D in the revised manuscript.

**5. The overall reduction in viral titer is modest for BET1 and greater for USE1 in Figure 4E, but in both cases there is viral propagation, though the title suggests USE 1 is “essential” for viral propagation. The title should be revisited to more accurately reflect the data, and the discussion should acknowledge this.**

Thank you very much for this important comment. We agree with the reviewer’s opinion that our initial title of “SNARE protein USE1 is essential for the glycosylation of mumps virus fusion protein and viral propagation” was not accurate. We have changed the role of USE1 in the title from “essential” to “important” and also made a similar statement in the discussion, in lines 329–446 of the revised manuscript.

**6. The data with integrin β in Figure 5E indicates that USE1 plays a role in complex carbohydrate addition to more membrane proteins than just mumps F, likely reflecting a key role for USE1 in trafficking of either the proteins that are modified or trafficking of components involved in glycan modification. Thus, USE1 appears to be a broadly-important host component, rather than one specifically involved in mumps F maturation – but this is not really reflected in the discussion.**

Thank you very much for this important comment. To address the issue raised by the reviewer, we have modified the main text in lines 309–311 of the revised manuscript.

**7. The modification of N-glycans is cell type dependent, so it is important that the findings be examined in more than one cell type – especially as the effect of USE1 knock-down varied between A549 and Vero cells.**

In accordance with the reviewer’s suggestion, we have evaluated the glycosylation of the F protein in USE1-knockdown A549 and Vero cells. As shown in Fig 6H of the revised manuscript, the expression of F protein in A549 cells was significantly decreased by USE1-knockdown, whereas the expression level and the glycosylation of F protein in Vero cells were not affected by USE1-knockdown. These findings demonstrate that the function of USE1 in the process of F protein maturation is cell-type dependent and that rather than being directly involved in glycosylation, USE1 is an important factor in the acquisition of F protein function through regulation of the early secretory pathway. Your comments have allowed us to properly evaluate the functionality of USE1, and we greatly appreciate it. We have added a description and discussion in lines 264–269, 329–346, and 796–798 of the revised manuscript.

**8. The authors suggest that USE1 is the first host factor identified as being involved in glycosylation of a paramyxovirus F protein – but this is completely incorrect. As stated earlier, N-linked glycosylation is a highly conserved process involving a set of enzymes in the ER which add the first high-mannose carbohydrate to the site. Further modifications are then carried out later by other identified host proteins. All of these factors are involved in F protein N-linked carbohydrate modification. In fact, analysis of viral glycoprotein modifications was a key part of identifying all of these components.**

Thank you very much for this important comment. We agree with the reviewer’s comment that our statement “USE1 is the first host factor identified as being involved in glycosylation of a paramyxovirus F protein” in the discussion section of the initially reviewed manuscript was incorrect. We have changed the word “first” to “new” on line 350 in the discussion of the revised manuscript.

**In addition, the change observed does not verify that USE1 actually is involved in glycosylation - or more accurately modification of the glycan chains. It suggests that USE1 is playing a role in trafficking (consistently with its cellular function) that impacts modification of the carbohydrate chains, potentially by increasing or decreasing the resident time of mumps F in a portion of the Golgi, or in trafficking cellular glycan-modifying enzymes. In fact, given the cytosolic localization of USE1, it would not be available to directly act on the N-linked carbohydrates in mumps F.**

Thank you very much for this important comment. We agree with the reviewer’s comment. To address this issue, we have modified the lines 303–311 in the main text of the revised manuscript.
